# In-Orbit Optimal Safe Formation Control for Surrounding an Unknown Huge Target with Specific Structure by Using Relative Sensors Only

**DOI:** 10.3390/s25175606

**Published:** 2025-09-08

**Authors:** Bosong Wei, Cong Li, Zhaohui Dang, Xiaokui Yue

**Affiliations:** 1 School of Astronautics, Northwestern Polytechnical University, 127 Youyi West Road, Xi’an 710072, China; weibosong9589@163.com (B.W.); licong1@mail.nwpu.edu.cn (C.L.); 2 National Key Laboratory of Aerospace Flight Dynamics, Northwestern Polytechnical University, Xi’an 710072, China

**Keywords:** formation surrounding control (FSC), escaping target, collision avoidance, differential game, satellite formation control

## Abstract

**Highlights:**

**What are the main findings?**

- User-defined boundaries without any initial conditions are used to directly shape the tracking performance of the unknown target, which only asks for relative position and velocity sensors.
- Based on a skillful collision threat modeling technique called the ACSB envelope, optimal strategies and trajectories are found for each satellite, which ensures collision avoidance for the arbitrary satellite with respect to all other satellites and all specific structures of the huge target.

**What is the implication of the main finding?**

- Optimal surrounding control by low-cost satellite formation of unknown huge target with non-ignorable specific structures.

**Abstract:**

The issue of in-orbit optimal safe surrounding control for service satellite (SSat) formation against a huge unknown target satellite (TSat) with specific structures is solved by using relative measurements only, and an optimal cooperative safe surrounding (OCSS) hybrid controller achieving both target tracking (TT) and configuration tracking (CT) is proposed corresponding to the two equal sub-objectives. Facing the challenges caused by incomplete information of the TSat, by using relative measurements only, the initial-condition-free boundaries are constructed by an arctan-based state transformation to directly constrain the target tracking error to perform prescribed transient and steady-state behaviors. Based on the shared TT control law, optimal collision-free CT controllers for all SSats are further solved via a nonzero-sum differential game, where the collision threat from all SSats and target structures are modeled by a novel circumscribed-sphere model. Finally, the effectiveness and advantages of the proposed OCSS control technique is verified by simulation results.

## 1. Introduction

As one of the typical and important space operation forms, multi-satellite formation has played an important role in space tasks. Furthermore, formation surrounding control (FSC), which may also be known as target enclosing/encirclement control (TEC) or target guarding control (TGC), using multi-satellite formation has become an inevitable and necessary operation for several state-of-the-art space tasks such as the pursuit-escape (PE) issue [1,2], proximity detection [3,4], collaborative takeover [5,6], attitude detumbling [7,8] and so on [9,10]. For proximity operation tasks, it is necessary to find optimal safe trajectories for multiple satellites to keep enough distance from both each other and the huge target and its specific structures. For this purpose, keeping the formation tracking the target is the most important technique for FSC tasks.

For the formation tracking task with ideal conditions, several attractive solutions that achieve cooperative in-orbit target tracking have been given. For example, Nash equilibrium strategies are given for each satellite by [11] to overcome communication delays and uncertainties occurring in cooperative target containment. A distributed formation tracking controller is designed by [12] with angular velocity constraints under partly known information. However, escaping non-cooperative targets presents great challenges for the aforesaid formation tracking methods. As a basic idea, zero-sum games are commonly used to handle the target tracking or capturing tasks. Considering a non-cooperative target with a stronger maneuver capability, pursuit controllers and capturing conditions for multiple pursuers are given in [13]. Optimal tracking strategies for the target are derived by the MPC framework in [14] based on zero-sum cost functionals. A target-behavior-free tracking controller for pursuers with interception angle constraints is proposed in [15] through a zero-sum differential game. In these game-based situations, the target can obey a unified cost functional with all pursuers, and thus, it escapes along a given saddle-point strategy, namely the “worst impact” trajectories. However, in some possible cases, the behavior principle of these non-cooperative targets may not be priori information, which forms incomplete-information game cases.

Achieving formation tracking control against a target with both unknown dynamics and escaping behaviors can be divided into two types. Depending on the introduction of extra estimators is one of them. The target dynamics is estimated by using range measurements by only [16]. A partly unknown situation is considered in [17], and an adaptive optimal PE strategy is given. Working as an alternative solution, several estimator-free techniques are also studied. The discrete difference equation is used in [18] to design a model-free multi-satellite surrounding control scheme. As a direct method, several barrier function-based control methods are proposed in [19,20,21,22,23] to constrain the system output directly, of which the dynamics is completely unknown.

The consideration on the safety of the operation formation is not enough by the approaches above. As a basic safety requirement, collision avoidance is necessary for satellite flying, especially for the satellite formation tackling surrounding tasks against a huge target with specific structures, where collision threats are mainly caused by two aspects, namely the one from the other formation satellites and the one from the target structures. Collision avoidance between each formation satellite and each other has been widely discussed by several recent works. A survey of spacecraft formation control with collision avoidance using hall thrusters is given in [24]. From this, a basic approach for collision avoidance is an artificial potential function (APF) [25] due to its convenient implementation and low computational load. An adaptive safe cooperative controller is given in [26] for satellite formation with uncertainties, and the desired formation configuration is guaranteed while the collision among other satellites is avoided. Also considering optimization requirements, optimal solutions for collision-free FSC considering presetting performance is given by [27] through a differential game framework.

On the other hand, tackling collision avoidance with irregular obstacles faces a bigger challenge, since distance assessment from satellites to obstacle surface suffers significant difficulties. The existing collision-avoidance techniques facing irregular obstacles can be mainly classified into two categories, including path planning [28,29] and reactive control [30,31,32]. Especially for general non-spherical obstacles, which can be commonly abstracted from specific structures of the huge space target, a repulsive potential field is designed for irregular celestial bodies in [33]. As for non-spherical obstacles with simple shapes, and an artificial potential field based on a super ellipsoid is given in [34] to approximate the envelope of a cuboid or a cylinder. By changing different principles of parameter modeling, such a scheme can be suitable for pyramids, cones, trapezoids, and so on. Based on this, a spacecraft close-range proximity problem is studied in [35] by using the approximate distance from a point to ellipsoidal envelopes of the spacecraft. By simplifying the huge space target as a hub–beam system formed as a combination of two ellipsoids, a safe controller is designed in [32] for in-orbit assembly missions.

Based on all the relevant works above, the optimal FSC issue with collision avoidance from dynamic obstacles (including other formation satellites and all specific structures of the target) against an escaping unknown target is still open. An optimal safe collision-avoidance control law is designed for SSat formation to achieve FSC against a fully unknown huge target with specific structures by using relative measurements only. The main contributions are summarized as follows:

- Considering a TSat and that the dynamic properties and escaping behavior of the TSat are both unknown, the studied FSC issue is decoupled into a TT issue and a CT issue, and it results in a convenient hybrid controller structure called an OCSS where the TT controller and the CT controller can be designed independently. Compared with the existing estimation-based FSC methods, estimators or approximators are not asked by the proposed TT technique, which reduces the complexity of the control system.
- An effective TT is achieved by using funnel-like boundaries to directly shape the transient behaviors to show a prescribed performance. The proposed funnel-like boundaries are initial-condition-free. Such boundaries are extended from the existing initial-dependent boundaries by a new transformation. As a result, the initial-condition-free boundaries can achieve effective constraint of the arbitrary initial tracking error, and thus, the singularity issue caused by the large initial error can be completely avoided.
- An adjustable cube-segmentation-based (ACSB) modeling method is proposed for specific structure modeling, which achieves an approximate but convenient envelope modeling method with variable accuracy. Furthermore, optimal safe surrounding strategies with two parts of the collision threats being avoided are given via a nonzero-sum game.

The rest of the paper is organized as follows. In Section 2, the surrounding task studied is equivalently formulized into two sub-objectives while the collision threat during the surrounding configuration tracking is described as tunning costs. Then, the design and stability analysis of the safe surrounding controller are designed in Section 3 and Section 4, respectively. Finally, in Section 5, the effectiveness and advantages of the proposed techniques are clarified by the simulation results.

Notation. The following notations are used throughout this paper. Vector $\left|S\right|$ is obtained from the absolute value of each element of vector s, and the operation $s^{2}≜s^{T}s$. ${\left[\cdot \right]}^{T}$ denotes the transpose of a vector or a matrix ·. R and $N^{+}$ denote the set of real numbers and positive integer numbers, respectively. $R^{n}$ and $R^{n\times m}$ denote the n-dimensional vector and n×m matrix, respectively.

## 2. Problem Formulation

**Figure 1:**
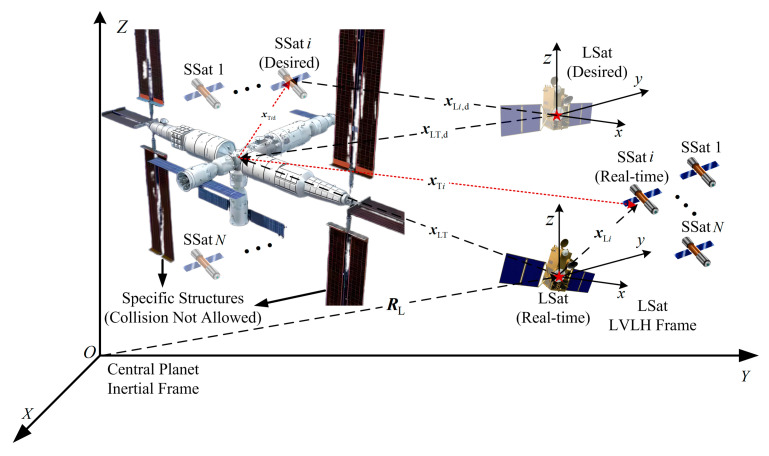
Close-range surrounding task description against a TSat with a specific structure by using N SSat formation under the command of a LSat.

A close-range surrounding task against an unknown non-cooperative TSat with a specific structure by N SSats driven by a leader satellite (LSat) is considered, as shown in Figure 1. The dynamic properties (including mass and orbital parameters) and maneuver commands of the TSat are all infeasible to both the LSat and all SSats, and these are the unknown elements considered for the non-cooperative TSat in this paper. The relative vectors (including the relative position and velocity) and shape parameters (including TSat size and the appearance parameters of the specific structures) of the TSat can be measured by the LSat accurately. Thus, to achieve a close-range surrounding of the TSat, all SSats are desired to form and track an expected close-range configuration, which is distributed in the gap of the TSat real structure, namely, $\left\|{\tilde{x}}_{Ti}\right\|≜\left\|x_{Ti}-x_{Ti,d}\right\|<\varepsilon_{Ti}$ hold for all i=1,…,N with $\varepsilon_{Ti}$ being a positive bound constant as shown in Figure 1. Each $x_{Ti}={\left[q_{Ti}^{T},{\dot{q}}_{Ti}^{T}\right]}^{T}\in R^{6}$ represents the relative vector from the TSat to SSat i, and $x_{Tid}={\left[q_{Ti,d}^{T},{\dot{q}}_{Ti,d}^{T}\right]}^{T}\in R^{6}$ represents the desired one. Furthermore, collision avoidance for all SSats and all specific structures of the huge target is asked.

**Remark 1.** 
*The close-range surrounding task studied in this paper is achieved by using N SSats under the command of one LSat. Different from the other FSC mission by using multiple homogeneous satellites with equal capacities [16,17], the scheme used in this paper will be more suitable for low-cost service formation, since the necessary measurement and calculation for unknown target tracking are now required only on the LSat instead of each SSats, and the TT control command calculated by the LSat can be directly shared to all SSats.*

### 2.1. Equivalent Formation Surrounding Modeling

Noting all $x_{Ti}$ in Figure 1 may be infeasible for low-cost SSats without specific sensors. In this section, the aforesaid objective is divided to be achieved by two parts equivalently. By setting a LSat, the following state equations can be obtained:

(1) $$\begin{array}{l} x_{Ti}=\left(-x_{\mathrm{LT}}\right)+x_{Li}=x_{\mathrm{TL}}+x_{Li}\\ x_{Ti,d}=\left(-x_{\mathrm{LT},d}\right)+x_{Li,d}=x_{\mathrm{TL},d}+x_{Li,d} \end{array}$$

From Equation (1), the dynamics of ${\tilde{x}}_{Ti}$ can be equivalently rewritten as follows:

(2) $$\begin{array}{l} {\dot{\tilde{x}}}_{Ti}≜{\dot{x}}_{Ti}-{\dot{x}}_{Ti,d}\\ \;=\left({\dot{x}}_{\mathrm{TL}}+{\dot{x}}_{Li}\right)-\left({\dot{x}}_{\mathrm{TL},d}+{\dot{x}}_{Li,d}\right)\\ \;={\dot{\tilde{x}}}_{\mathrm{TL}}+{\dot{\tilde{x}}}_{Li} \end{array}$$

where the relative orbital state of the TSat is written as $x_{\mathrm{LT}}=-x_{\mathrm{TL}}={\left[q_{\mathrm{LT}}^{T},{\dot{q}}_{\mathrm{LT}}^{T}\right]}^{T}\in R^{6}$ with $q_{\mathrm{LT}}={\left[q_{\mathrm{LT}1},q_{\mathrm{LT}2},q_{\mathrm{LT}3}\right]}^{T}\in R^{3}$ and ${\dot{q}}_{\mathrm{LT}}=\left[{\dot{q}}_{\mathrm{LT}1},{\dot{q}}_{\mathrm{LT}2},{\dot{q}}_{\mathrm{LT}3}\right]\in R^{3}$ being the position vector and the velocity vector from the LSat to the TSat, respectively. The relative orbital state of each SSat i is written as $x_{Li}={\left[q_{Li}^{T},{\dot{q}}_{Li}^{T}\right]}^{T}\in R^{6}$ with $q_{Li}\in R^{3}$ and ${\dot{q}}_{Li}\in R^{3}$ being the position vector and the velocity vector from the LSat to the TSat, respectively. Considering the desired states for $x_{\mathrm{LT}}$ and $x_{Li}$ as $x_{\mathrm{LT},d}={\left[q_{\mathrm{LT},d}^{T},{\dot{q}}_{\mathrm{LT},d}^{T}\right]}^{T}$ and $x_{Li,d}={\left[q_{Li,d}^{T},{\dot{q}}_{Li,d}^{T}\right]}^{T}$, the CT error can be defined as ${\tilde{x}}_{Li}={\left[{\tilde{q}}_{Li}^{T},{\dot{\tilde{q}}}_{Li}^{T}\right]}^{T}\in R^{6}$ with ${\tilde{q}}_{Li}≜q_{Li}-q_{Li,d}$ and ${\dot{\tilde{q}}}_{Li}≜{\dot{q}}_{Li}-{\dot{q}}_{Li,d}$, and the TT error of SSat i can be defined as ${\tilde{x}}_{\mathrm{TL}}={\left[{\tilde{q}}_{\mathrm{TL}}^{T},{\dot{\tilde{q}}}_{\mathrm{TL}}^{T}\right]}^{T}\in R^{6}$ with ${\tilde{q}}_{\mathrm{TL}}≜q_{\mathrm{TL}}-q_{\mathrm{TL},d}$ and ${\dot{\tilde{q}}}_{\mathrm{TL}}≜{\dot{q}}_{\mathrm{TL}}-{\dot{q}}_{\mathrm{TL},d}$. As a result, all $x_{Ti}$ can be equally calculated by $x_{\mathrm{LT}}$ and $x_{Li}$ which are all feasible knowledges for the LSat and all SSats, respectively.

According to Equation (2), by Euler-Lagrange equation [36], the relative motion of TSat and SSat i referring to the LSat satisfy the following differential equations.

(3) $$\begin{array}{l} {\dot{x}}_{\mathrm{LT}}\left(t\right)={\left[{{\dot{q}}_{\mathrm{LT}}}^{T},{{\ddot{q}}_{\mathrm{LT}}}^{T}\right]}^{T}\\ \;=\left[\begin{array}{c} {\dot{q}}_{\mathrm{LT}}\\ -\hat{C}\left(\omega \right){\dot{q}}_{\mathrm{LT}}-N_{\mathrm{LT}}\left(q_{\mathrm{LT}}\right)+\frac{u_{T}}{m_{T}}-\frac{u_{L}}{m_{L}} \end{array}\right] \end{array}$$

(4) $$\begin{array}{l} {\dot{x}}_{Li}\left(t\right)={\left[{{\dot{q}}_{Li}}^{T},{{\ddot{q}}_{Li}}^{T}\right]}^{T}\\ \;=\left[\begin{array}{c} {\dot{q}}_{Li}\\ -\hat{C}\left(\omega \right){\dot{q}}_{Li}-N_{Li}\left(q_{Li}\right)+\frac{u_{i}}{m_{i}}-\frac{u_{L}}{m_{L}} \end{array}\right] \end{array}$$

where $u_{T}={\left[u_{T1},u_{T2},u_{T3}\right]}^{T}\in R^{3}$ is the unknown maneuver command of the TSat. $u_{L}={\left[u_{L1},u_{L2},u_{L3}\right]}^{T}\in R^{3}$ and $u_{i}={\left[u_{i1},u_{i2},u_{i3}\right]}^{T}\in R^{3}$ denote the active control commands of the LSat and SSat, respectively. $m_{T}\in R$, $m_{L}\in R$, and $m_{i}\in R$ represent the mass of the TSat, LSat, and each SSat i. Considering the absolute orbital angular velocity of the LSat $\omega \left(t\right)\in R$ and the absolute position of the LSat $R_{L}\in R^{3}$ in the central planet inertial frame, $\hat{C}\left(\omega \right)\in R^{3\times 3}$ and nonlinear terms $N_{\mathrm{LT}}\left(q_{\mathrm{LT}}\right)\in R^{3}$, $N_{Li}\left(q_{Li}\right)\in R^{3}$ can be obtained [27].

**Assumption 1.** 
*For the TSat described by Equation (3), $m_{T}$ and $u_{T}$ are unknown information while $x_{\mathrm{LT}}={\left[q_{\mathrm{LT}}^{T},{\dot{q}}_{\mathrm{LT}}^{T}\right]}^{T}$ can be accurately measured by the LSat. The unknown maneuver command of the TSat is bounded, namely $u_{T}={\left[u_{T1},u_{T2},u_{T3}\right]}^{T}$ satisfies that $u_{Tl}\leq {\bar{u}}_{T}$, l=1,2,3, with ${\bar{u}}_{T}$ being an unknown positive constant.*

Further, all ${\tilde{x}}_{Li}$ satisfy the following state space dynamics:

(5) $$\begin{array}{l} {\dot{\tilde{x}}}_{Li}=\bar{A}\left(\omega \right)\left({\tilde{x}}_{Li}+{\dot{\tilde{x}}}_{Li,d}\right)-\bar{B}f_{i}\\ \;+{\bar{H}}_{i}^{-1}\bar{B}u_{i}-{\bar{H}}_{L}^{-1}\bar{B}u_{L} \end{array}$$

where $f_{i}\left(t\right)=\mu_{c}(q_{Li}+re_{y})/{\left\|R_{L}+q_{Li}\right\|}^{3}\in R^{3}$ with $\mu_{c}\in R$ being the gravitational constant, $r\left(t\right)≜\left\|R_{L}\left(t\right)\right\|\in R$ being the position vector of the LSat in the central planet inertial frame, constant vector $e_{y}≜{\left[0,1,0\right]}^{T}\in R^{3}$, inertia matrix of SSat i ${\bar{H}}_{i}=m_{i}I_{6}$, and inertia matrix of the LSat ${\bar{H}}_{L}=m_{L}I_{6}$ with $I_{6}\in R^{6\times 6}$ being the identity matrix. $\bar{B}={\left[0_{3},I_{3}\right]}^{T}\in R^{6\times 3}$. Since $f_{i}\left(t\right)$ are Lipschitz continuous, here exists a matrix-value function ${\bar{F}}_{i}\left(\cdot \right)\in R^{6\times 6}$, ensuring ${\bar{F}}_{i}\left({\tilde{x}}_{Li}\right){\tilde{x}}_{Li}=\bar{A}{\tilde{x}}_{Li}-\bar{B}f_{i}\left(t\right)$ [37]. Based on Equation (2), the controller design of SSat i can be divided as follows:

(6) $$u_{i}=u_{Li}+u_{ci}+u_{id}$$

where the term $u_{Li}≜{\bar{B}}^{†}{\bar{H}}_{i}{\bar{H}}_{L}^{-1}\bar{B}u_{L}\in R^{3}$ is used to make a synchronous maneuver at SSat i according to the commands shared by the LSat. $u_{ci}\in R^{3}$ for all i=1,…,N are used to form and stably track the desired surrounding configuration. From this perspective, the safe formation surrounding the control design for all SSats can be completely and equivalently decoupled into two independent parts, namely, the TT part and surrounding CT part.

**Remark 2.** 
*The FSC issues directly solved by the zero-sum differential game framework [13,14,15] commonly assume that all pursuers and escapees share a unified known cost functional, which can obtain a saddle-point strategy for the pursuer under the worst impact caused by the escapee. Inspired by [17], such a prerequisite may not be satisfied by some specific non-cooperative targets. Equation (6) shows a decoupling mechanism, which divides the whole studied FSC task into two parts while the controller of these two parts can be designed independently.*

### 2.2. Collision Threat Modeling During FSC Tasks

**Figure 2:**
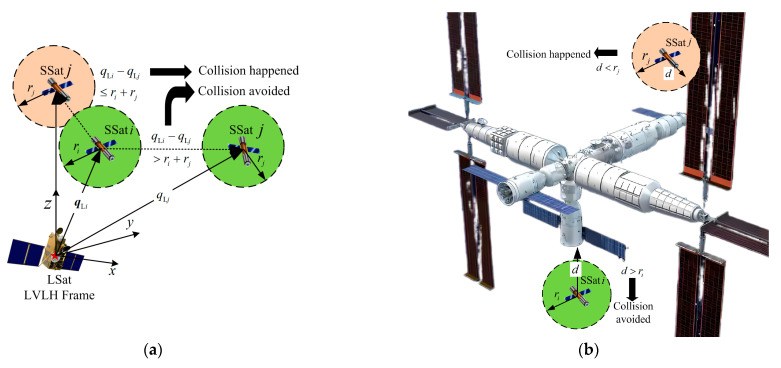
The potential collision threat surrounding a target with specific structures. (**a**) Collision between two SSats. (**b**) Collision between SSats and specific structures.

This section focuses on an appropriate formulation of collision threats for all SSats, which is necessary for collision-free controller design. Considering the close-range surrounding requirements against the TSat with specific structures, as shown in Figure 1, the potential collision threat of SSat i during the studied target surrounding process are mainly two parts, namely the threat from all specific structures of the huge target and the threat from each SSat j, j≠i, as shown in Figure 2.

To describe the threat from other SSats shown in Figure 2a, a circumscribed sphere with safe radius $r_{i}$ can be directly used for each SSat. Thus, the collision threat imposed by SSat j, j≠i, to SSat i can be written as follows.

(7) $$c_{\mathrm{FC},i}\left(\tilde{x}\right)=\sum_{\begin{array}{l} j=1\\ j\neq i \end{array}}^{N}\frac{1}{{\left({\left\|\left({\tilde{q}}_{Li}+q_{Li,d}\right)-\left({\tilde{q}}_{Lj}+q_{Lj,d}\right)\right\|}^{2}-{\left(r_{i}+r_{j}\right)}^{2}\right)}^{h_{\mathrm{FC}}}}$$

where $c_{\mathrm{FC},i}\left(\tilde{x}\right)$ is a scalar function with a positive correlation to the collision threat between SSat i and SSat j, j≠i. $h_{\mathrm{FC}}\geq 1$ adjusts the sensitivity to the collision threat.

To describe the collision threat from huge structures with possibly irregular profiles, the mass-point model is indeed not enough and using circumscribed spheres of the whole specific structures directly thoroughly occupies the reachable space of SSats. Inspired by the conclusion that the smallest enveloping sphere of a cube is its circumscribed sphere, an adjustable cube-segmentation-based (ACSB) method is proposed to give a complete envelope of the huge TSat with a specific structure, and a cross section of standard cone is used as an example to explain the mechanism of the proposed ACSB envelope modeling.

**Figure 3:**
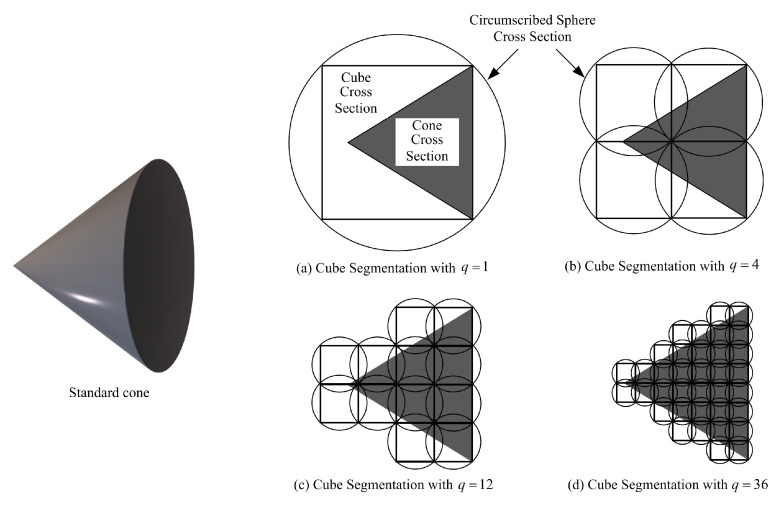
Mechanism of the proposed ACSB envelope modeling with different sizes of cube segmentation.

As shown in Figure 3, the sample cone can be always contained by q cubes with q≥1, $q\in N^{+}$. Considering $C_{g}$ being the circumscribed sphere of the gth cube with radius $r_{g}$ and $V_{C_{g}}$ being the circumscribed-sphere volume of the gth cube, the larger the side length of the gth cube, the larger the total enveloping volume of the circumscribed spheres $\sum_{g=1}^{q}V_{C_{g}}$. As a result, by selecting different cube sizes for each specific structure of the TSat, the accuracy of the envelope modeling can be flexibly adjusted.

**Figure 4:**
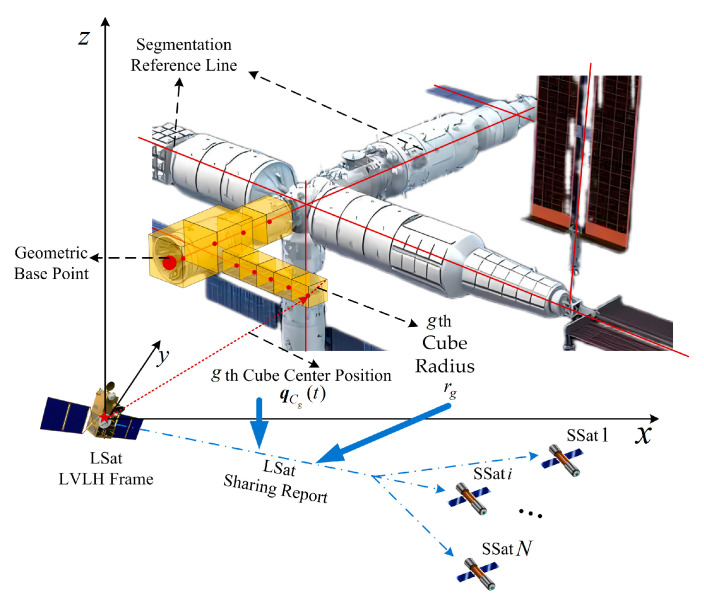
Implementation process diagram of ACSB-based target cube segmentation and information sharing by the LSat.

Similarly to the modeling process shown in Figure 3, the envelope of the huge TSat with specific structures can be finally modeled as a circumscribed-sphere set $\Omega_{C}$ with different cube sizes, namely $\Omega_{C}≜\left\{C_{1},\ldots ,C_{g},\ldots ,C_{q}\right\}$ with $C_{1}$ and $C_{q}$ being the circumscribed sphere of the 1st and the qth cube, as shown in Figure 4. Since the non-cooperative target is time-varying with respect to the LSat, $q_{C_{g}}(t)$ is the real-time geometric center position of the gth cube (also its circumscribed sphere) in the LSat LVLH frame. Both $q_{C_{g}}(t)$ and $r_{g}$ of each cube are finally reported by the LSat to all SSats as shared information.

Further, based on the ACSB envelope modeling shown in Figure 3, the collision threat caused by the gth cube for each SSat i can be written as follows:

(8) $$c_{C_{g},i}\left(q_{Li}\right)=\sum_{g=1}^{q}\frac{1}{{\left({\left\|q_{Li}-q_{C_{g}}\right\|}^{2}-{\left(r_{i}+r_{g}\right)}^{2}\right)}^{h_{C_{g},i}}}$$

where $c_{C_{g},i}\left(x_{Li}\right)$ is a scalar function with a positive correlation to the collision threat between SSat i and the gth circumscribed sphere. $h_{C_{g},i}\geq 1$ adjusts the sensitivity to the collision threat.

**Assumption 2.** 
*The attitude of the TSat can be ignored. The TSat’s shape parameters of specific structures can be accurately measured by the LSat, and the cube segmentation of the huge target structures can be achieved via the ACSB method by the LSat, and the parameters of all the circumscribed spheres can be shared to each SSats with no time delay.*

**Assumption 3.** 
*The initialization of the SSats formation is asked to be collision-free, namely $\left\|q_{Li}-q_{Lj}\right\|>r_{i}+r_{j}$ and $\left\|q_{Li}-q_{C_{g}}\right\|>r_{i}+r_{g}$ for all i=1,…,N at t=0.*

**Assumption 4.** 
*The cube segmentation of the huge target structures is asked to satisfy that there always exists a feasible trajectory from real-time position $q_{Li}$ for all i=1,…,N to the desired configuration point $q_{Li,d}$.*

**Remark 3.** 
*Compared with the existing accurate collision threat modeling methods based on [4,38], the proposed ACSB-based collision threat modeling method requires an approximate for the TSat structural envelope, which can effectively reduce the measurement load to assess the collision threat from the TSat. Thus, it is more suitable for low-cost SSat formations, although the reachable domain may be sacrificed. The cube segmentation resolution, namely the envelope modeling precision, can be adjusted according to mission requirements.*

**Remark 4.** 
*Cube segmentation is finished through an off-line process by the LSat. Although the number of cube segmentation is adjustable for different task requirements, it remains constant during the whole FSC process. The calculation load of the ACSB-based modeling are mainly two parts, namely the load for the LSat and the load for all SSats. The calculation load the ACSB-based modeling aims to reduce is mainly for the latter, since the online calculation load for each SSat is only the simple norm calculation in (8) by using the information shared by the LSat, and the calculation times in each control step is equal to the number of cubes.*

### 2.3. Game-Based Description for Collision-Free CT Problem

To find optimal collision-free trajectories for all SSats to form the desired surrounding configuration around the huge target, based on the collision threat modeled above, the collision-free CT problem is described as a nonlinear nonzero-sum differential game.

Considering an integrated CT error $\tilde{x}≜{\left[{\tilde{x}}_{L1}^{T},\ldots ,{\tilde{x}}_{Li}^{T},\ldots ,{\tilde{x}}_{LN}^{T}\right]}^{T}\in R^{6N}$, substituting Equation (6) into Equation (5), the dynamics of $\tilde{x}$ can be directly obtained as follows:

(9) $$\begin{array}{l} \dot{\tilde{x}}=A\left(\tilde{x}\right)\tilde{x}+H^{-1}B_{i}u_{ci}\\ \;+\sum_{\begin{array}{l} j=1\\ j\neq i \end{array}}^{N}H^{-1}B_{j}u_{cj} \end{array}$$

with

(10) $$\left\{\begin{array}{l} \begin{array}{l} A\left(\tilde{x}\right)≜\mathrm{diag}\left({\bar{F}}_{1},\ldots ,{\bar{F}}_{i},\ldots ,{\bar{F}}_{N}\right)\in R^{6N\times 6N}\\ H≜I_{N}⊗{\bar{H}}_{i\left(j\right)}\in R^{6N\times 6N} \end{array}\\ B_{i\left(j\right)}≜{\left[0_{3\times 6}^{T},\ldots ,{\left({\bar{B}}^{T}\right)}_{\left(i\left(j\right)\right)},\ldots ,0_{3\times 6}^{T}\right]}^{T}\in R^{6N\times 3} \end{array}\right.$$

Considering the collision threat between all SSats and all specific structures as competitive factors, the nonlinear cost functional $J_{i}$ of each SSat i is designed as follows:

(11) $$\begin{array}{l} J_{i}\left(\tilde{x},u_{c1},\ldots ,u_{cN}\right)≜\\ \;\frac{1}{2}\int_{0}^{\infty }\left(q_{i}(\tilde{x})+f_{i}\left(u_{c1},\ldots ,u_{cN}\right)\right)dt\\ f_{i}\left(u_{c1},\ldots ,u_{cN}\right)≜u_{ci}^{T}u_{ci}+\sum_{\begin{array}{l} j=1\\ j\neq i \end{array}}^{N}u_{cj}^{T}u_{cj} \end{array}$$

where $f_{i}\left(u_{c1},\ldots ,u_{cN}\right)$ is designed to guarantee minimum control consumption. The nonlinear term $q_{i}(\tilde{x})$ is designed as follows:

(12) $$q_{i}\left(\tilde{x}\right)=\left(\kappa_{i}+\alpha_{\mathrm{FC},i}c_{\mathrm{FC},i}+\alpha_{C_{g},i}c_{C_{g},i}\right){\tilde{x}}_{Li}^{T}{\tilde{x}}_{Li}$$

where $\kappa_{i}$, $\alpha_{\mathrm{FC},i}$, and $\alpha_{C_{g},i}$ are proportional gain and positive weight coefficients, respectively, and the design of nonlinear term $c_{\mathrm{FC},i}$ and $c_{C_{g},i}$ are given in Equations (7) and (8), respectively.

According to Equations (9) and (11), the CT problem can be described as a nonzero-sum differential game. By the extremal conditions [6], solving the Nash equilibrium strategies $u_{ci}^{*}$ boils down to solving the following coupled Hamilton–Jacobi–Isaacs (HJI) partial differential equations (PDEs) with respect to the value function $V_{i}^{*}\left(\tilde{x}\right)$.

(13) $$\begin{array}{l} 0=\frac{\partial V_{i}^{*}\left(\tilde{x}\right)}{\partial \tilde{x}}\left(A\left(\tilde{x}\right)\tilde{x}+\sum_{i=1}^{N}H^{-1}B_{i}u_{ci}^{*}\right)+\\ \;\frac{1}{2}\left(q_{i}\left(\tilde{x}\right)+u_{ci}^{*T}R_{ii}u_{ci}^{*}+\sum_{j=1,j\neq i}^{N}u_{cj}^{*T}R_{jj}u_{cj}^{*}\right) \end{array}$$

**Remark 5.** 
*Thanks to the collision threat for specific structures and each SSat i, the total collision threat of SSat i can be determined by both Equations (8) and (12). According to Assumption 3, the initialization ensures that the collision threat of each SSat i is bound but not equal to zero. As running costs for the differential game are determined by Equations (9) and (11), a Nash equilibrium is called to be achieved if and only if $q_{i}\left(\tilde{x}\right)$ in Equation (11) are minimized, which avoids the collision between all SSat i with respect to arbitrary target specific structures and arbitrary SSat j, j≠i. More detailed proof can be found in [39].*

### 2.4. Control Objectives and Preliminaries

As the collision-free formation surrounding the control design for all SSats is decoupled into the TT part and the CT part according to Equations (2) and (6), based on all formulation analyses above, the objectives $\left\|{\tilde{x}}_{Ti}\right\|≜\left\|x_{Ti}-x_{Ti,d}\right\|<\varepsilon_{Ti}$ can be finally achieved by the following objectives:

**Problem 1.** 
Consider relative motion dynamics of the TSat and SSat i in Equations (3) and (4). Based on Equations (2) and (6), the safe formation surrounding control against the unknown huge TSat with specific structures boils down to a TT controller $u_{L}$ design for the LSat and Nash equilibrium CT controller’s $u_{ci}^{*}$ design for all SSats, which guarantees that the closed-loop TT error ${\tilde{x}}_{\mathrm{TL}}$ and the closed-loop CT error ${\tilde{x}}_{Li}$ are both bound while avoiding collisions, namely $\left\|{\tilde{x}}_{\mathrm{LT}}\right\|≜\left\|x_{\mathrm{LT}}-x_{\mathrm{LT},d}\right\|\leq \varepsilon_{\mathrm{LT}}$, $\left\|{\tilde{x}}_{Li}\right\|≜\left\|x_{Li}-x_{Li,d}\right\|\leq \varepsilon_{Li}$ with $\varepsilon_{\mathrm{LT}}$ and $\varepsilon_{Li}$ being positive bound constants while $\left\|q_{Li}-q_{Lj}\right\|>r_{i}$ and $\left\|q_{Li}-q_{C_{g}}\right\|>r_{i}+r_{g}$.

In what follows, several necessary definitions are given.

**Definition 1.** 
(Nash equilibrium strategies) [5] Consider a set U consisting of the strategy groups stabilizing the CT error system Equation (9). A group of strategies $\text{\{}u_{c1}^{*},\ldots ,u_{ci}^{*},\ldots ,u_{cN}^{*}\text{\}}\in U$ are said to be the Nash equilibrium strategies of the differential game determined by Equations (9) and (10) if and only if $J_{i}\left(\tilde{x},u_{c1}^{*},\ldots ,u_{ci}^{*},\ldots ,u_{\mathrm{cN}}^{*}\right)\leq J_{i}\left(\tilde{x},u_{c1}^{*},\ldots ,u_{ci},\ldots ,u_{\mathrm{cN}}^{*}\right)$ holds for any $\text{\{}u_{c1}^{*},\ldots ,u_{ci},\ldots ,u_{cN}^{*}\text{\}}\in U$ with $u_{ci}\neq u_{ci}^{*}$.

**Definition 2.** 
(Approximate Nash equilibrium strategies) A group of strategies $\text{\{}u_{c1}^{*},\ldots ,u_{ci}^{*},\ldots ,u_{cN}^{*}\text{\}}$ ∈U are said to be approximate Nash equilibrium strategies of the differential game determined by Equations (9) and (11) if and only if $J_{i}(\tilde{x},u_{c1}^{*},\ldots ,u_{ci}^{*},\ldots ,u_{cN}^{*})\leq J_{i}(\tilde{x},u_{c1}^{*},\ldots ,u_{ci},\ldots ,u_{cN}^{*})+{\overset{⌢}{e}}_{i}$ holds for any $\text{\{}u_{c1}^{*},\ldots ,u_{ci},\ldots ,u_{cN}^{*}\text{\}}\in U$ with $u_{ci}\neq u_{ci}^{*}$ and a scalar function ${\overset{⌢}{e}}_{i}(t)\geq 0$.

**Definition 3.** 
*(Diagonal matrix solutions $P_{i}$) [27] Consider the integrated CT error system Equation (9) and the individual cost functionals Equation (11). For existing matrix functions $\Sigma_{i}\left(\cdot \right):R^{6N}\to R^{6N\times 6N}$ satisfying $\Sigma_{i}\left(\cdot \right)+\Sigma_{i}^{T}\left(\cdot \right)>0$, $\Sigma_{i}\left(0\right)+\Sigma_{i}^{T}\left(0\right)>0$ and $Q_{i}\left(\tilde{x}\right):R^{6N}$$\to R^{6N\times 6N}$ satisfying $q_{i}\left(\tilde{x}\right)={\tilde{x}}^{T}Q_{i}\left(\tilde{x}\right)\tilde{x}$. Then, a group of diagonal matrix functions $P_{i}\left(\cdot \right)=P_{i}^{T}\left(\cdot \right)$ are said to be diagonal matrix solutions of the HJI PDE Equation (13). if the following conditions are satisfied.*

*(1) For all *$\tilde{x}\in R^{6N}$, $P_{i}\left(\tilde{x}\right)$* satisfies the following:*

(14) $$\begin{array}{l} 0=P_{i}\left(\tilde{x}\right)A+A^{T}P_{i}\left(\tilde{x}\right)+Q_{i}\left(\tilde{x}\right)-\\ \;P_{i}\left(\tilde{x}\right)H^{-1}\left(B_{i}-\Delta B_{i}\right){\hat{B}}_{i}^{T}H^{-T}P_{i}\left(\tilde{x}\right)-\\ \;2\sum_{j=1,j\neq i}^{N}P_{i}\left(\tilde{x}\right)H^{-1}B_{j}{\hat{B}}_{j}^{T}H^{-T}P_{j}\left(\tilde{x}\right)-\\ \;\sum_{j=1,j\neq i}^{N}P_{j}\left(\tilde{x}\right)H^{-1}{\hat{B}}_{j}{\hat{B}}_{j}^{T}H^{-T}P_{j}\left(\tilde{x}\right)+\Sigma_{i}\left(\tilde{x}\right) \end{array}$$

*(2) Equation (14) holds for all *i=1,…,N*, *$\tilde{x}=0$.

*where *${\hat{B}}_{i}≜B_{i}+\Delta B_{i}$, $\Delta B_{i}={\left[0_{3\times 6},\ldots ,{\left(\Delta {\bar{B}}_{i}^{T}\right)}_{\;\;(i)},\ldots ,0_{3\times 6}\right]}^{T}\in R^{6N\times 3}$, $\Delta {\bar{B}}_{i}=[\Delta e_{1},\Delta e_{2},\Delta e_{3}]\in R^{6\times 3}$, *and*$\Delta e_{l}\in R^{6}$.

## 3. Main Results

Corresponding to the above controller objectives, an OCSS controller is given as follows.

Facing the challenges caused by unknown dynamics and maneuver commands, arctan-based transformation with respect to the measured TT error ${\tilde{q}}_{\mathrm{TL}}^{T}$ and ${\dot{\tilde{q}}}_{\mathrm{TL}}^{T}$ are designed as follows:

(15) $$\begin{array}{l} e_{1}=\frac{I_{3}}{k_{1}(t)}\arctan\left(q_{\mathrm{TL}}-q_{\mathrm{TL},d}\right)\\ e_{2}=\frac{I_{3}}{k_{2}(t)}\arctan\left({\dot{q}}_{\mathrm{TL}}-{\dot{q}}_{\mathrm{TL}}^{v}\right) \end{array}$$

where $k_{1}(t)=(1-\mu_{1})e^{-a_{1}t}+\mu_{1}$ and $k_{2}(t)=(1-\mu_{2})e^{-a_{2}t}+\mu_{2}$ are performance functions, which are designed to expand them into the shaping performance boundaries without initial conditions, which can directly constrain the output of $q_{\mathrm{TL}}$ and ${\dot{q}}_{\mathrm{TL}}$. $0<\mu_{r}<1$ and $a_{r}>0$, r=1,2. By using the transformed errors $e_{1}$ and $e_{2}$, virtual controller ${\dot{q}}_{\mathrm{TL}}^{v}$, and TT controller, $u_{L}$ can be obtained as follows:

(16) $${\dot{q}}_{\mathrm{TL}}^{v}=-\lambda_{1}I_{3}\tan e_{1}$$

(17) $$u_{L}=-\lambda_{2}I_{3}\tan e_{2}$$

where $\lambda_{1}$ and $\lambda_{2}$ are all positive parameters, which are designed later.

**Remark 6.** 
*Thanks to the skillful usage of the arctan-based transformation, compared with the existing whole-process constraint boundaries [40,41], initial-condition-free boundaries $\pm \tan(k_{r}(t)\pi /2)$ are constructed by Equation (15). Compared with the existing output-constrain-based methods [42,43], by adjusting $a_{r}$ and $\mu_{r}$, the boundaries constructed by Equation (15) achieve a whole-process constraint with adjustable performance. Meanwhile, noting that $q_{\mathrm{TL}l}(0)-q_{\mathrm{TL}l,d}(0)|$ $\left|<\tan(k_{1}(0)\pi /2\right)$ and $\left|{\dot{q}}_{\mathrm{TL}l}(0)-{\dot{q}}_{\mathrm{TL}l}^{v}(0)|<\tan(k_{2}(0)\pi /2\right)$ always hold, arbitrary bounded initial TT errors are constrained by the proposed boundaries, which implies that the controllers given in Equations (16) and (17) work globally.*

According to the individual cost functional design for each SSat i, a collision-free target surrounding control can be achieved by the Nash equilibrium of the nonzero-sum differential game defined by Equations (9) and (11). However, it is too difficult to solve the coupled HJI PDEs in Equation (13) analytically. Different from the existing methods, a constructable approximate value function $V_{i}\left(\tilde{x},\xi \right)$ with dynamic auxiliary variable $\xi =[\xi_{1}^{T},$ $\ldots ,\xi_{i}^{T},\ldots ,\xi_{N}^{T}{]}^{T}\in R^{6N}$ being immersed is designed for each SSat i as follows:

(18) $$\begin{array}{l} V_{i}\left(\tilde{x},\xi \right)=\\ \;\frac{1}{2}{\tilde{x}}^{T}P_{i}\left(\xi \right)\tilde{x}+\frac{1}{2}{\left(\tilde{x}-\xi \right)}^{T}R_{i}\left(\tilde{x}-\xi \right) \end{array}$$

where $R_{i}=R_{i}^{T}>0$ for all i=1,…,N are positive-definite coefficient matrices being designed later. According to the individual cost functionals in Equation (11), diagonal matrix solution $P_{i}\left(\cdot \right)$ satisfying Definition 1 is designed as follows:

(19) $$\begin{array}{l} P_{i}\left(\cdot \right)≜\left[\begin{array}{ccc} P_{11}^{i}\left(\cdot \right) & \ldots & P_{1N}^{i}\left(\cdot \right)\\ \vdots & \ddots & \vdots \\ P_{N1}^{i}\left(\cdot \right) & \ldots & P_{NN}^{i}\left(\cdot \right) \end{array}\right]\\ \left\{\begin{array}{l} \begin{array}{l} P_{pq}^{i}\left(\cdot \right)=\left(\sqrt{\vartheta_{i}\left(\cdot \right)}+\gamma_{i}\right)I,\;p=q=i\\ \vartheta_{i}\left(\cdot \right)≜\kappa_{i}+\alpha_{\mathrm{FC},i}c_{\mathrm{FC},i}\left(\cdot \right)+\alpha_{C_{g},i}c_{C_{g},i}\left(\cdot \right) \end{array}\\ P_{pq}^{i}\left(\cdot \right)=0,\;p\neq q,p=q\neq i \end{array}\right. \end{array}$$

To ensure all $V_{i}\left(\tilde{x},\xi \right)$ are minimized along their gradient direction, a tuning law is designed for the immersed auxiliary variable.

(20) $$\begin{array}{l} \dot{\xi }=-\eta {\sum_{i=1}^{N}\frac{\partial V_{i}}{\partial \xi }}^{T}\\ \;=-\eta \sum_{i=1}^{N}\left(\Psi_{i}^{T}\left(\tilde{x},\xi \right)\tilde{x}-R_{i}^{T}\left(\tilde{x}-\xi \right)\right) \end{array}$$

where $\Psi_{i}\left(\tilde{x},\xi \right)$ is the first-order partial-derivative matrix of vector $\left(1/2\right)\cdot P_{i}\left(\xi \right)\tilde{x}$. η∈R is a positive proportional coefficient to adjust the speed of the decent. Further, according to Equation (18), dynamic Nash equilibrium safe CT controllers $u_{ci}^{*}$ are given as follows.

(21) $$\begin{array}{l} u_{ci}^{*}=-\left(B_{i}^{T}+\Delta B_{i}^{T}\right)H^{-1}{\frac{\partial V_{i}}{\partial \tilde{x}}}^{T}\\ \;=-{\hat{B}}_{i}^{T}H^{-1}\left(P_{i}\left(\tilde{x}\right)\tilde{x}+\left(R_{i}-\Phi_{i}\right)\left(\tilde{x}-\xi \right)\right) \end{array}$$

where $\Phi_{i}\left(\tilde{x},\xi \right)$ is obtained by ${\left(\tilde{x}-\xi \right)}^{T}\Phi_{i}^{T}\left(\tilde{x},\xi \right)={\tilde{x}}^{T}\left(P_{i}\left(\tilde{x}\right)-P_{i}\left(\xi \right)\right)$. By all the designs above, we can obtain the following results.

**Theorem 1.** 
Consider the relative motion of the TSat and SSats in Equations (3) and (4). Describe the CT issue as the nonzero-sum game determined by the integrated system Equation (9) and individual cost functional Equation (11). Considering $R_{i}$ satisfying $R_{i}\sum_{j=1,\;j\neq i}^{N}R_{j}+{\left(R_{i}\sum_{j=1,\;j\neq i}^{N}R_{j}\right)}^{T}>0$ and tuning the auxiliary variable as in Equation (20), let the constructed $V_{i}\left(\tilde{x},\xi \right)$ satisfy $-\sum_{i=1}^{N}N\left(\left(\partial V_{i}/\partial \tilde{x}\right)H^{-1}{\hat{B}}_{i}{\hat{B}}_{i}^{T}H^{-T}{\left(\partial V_{i}/\partial \tilde{x}\right)}^{T}/2\right)-e_{i}\left(\tilde{x},\xi \right)/2\leq 0$ for the existing scalar function  $e_{i}(\tilde{x},\xi )≜$$-2I_{i}^{s}>0$. Then, by using the TT controller $u_{L}$ given in Equation (17) and the optimal CT controllers $u_{ci}^{*}$ given in Equation (21), we can obtain the following results.

*(i) (Optimality) Existing constant* $\bar{\eta }>0$ *and a compact set* $\Omega_{i}$ *containing the origin ensure that* $V_{i}\left(\tilde{x},\xi \right)$ *in Equation (18) satisfy the following inequalities.*

(22) $$\begin{array}{l} I_{i}^{s}≜\frac{\partial V_{i}}{\partial \tilde{x}}A\tilde{x}-\sum_{j=1,j\neq i}^{N}\frac{\partial V_{i}}{\partial \tilde{x}}H^{-1}B_{j}{\hat{B}}_{j}^{T}H^{-T}{\frac{\partial V_{j}}{\partial \tilde{x}}}^{T}-\\ \;\frac{1}{2}\sum_{i=1}^{N}\frac{\partial V_{j}}{\partial \tilde{x}}H^{-1}{\hat{B}}_{j}{\hat{B}}_{j}^{T}H^{-T}{\frac{\partial V_{j}}{\partial \tilde{x}}}^{T}-\\ \;\frac{1}{2}\frac{\partial V_{i}}{\partial \tilde{x}}H^{-1}\left(B_{i}-\Delta B_{i}\right){\hat{B}}_{i}^{T}H^{-T}{\frac{\partial V_{i}}{\partial \tilde{x}}}^{T}+\\ \;\frac{1}{2}q_{i}\left(\tilde{x}\right)+\frac{\partial V_{i}}{\partial \xi }\dot{\xi }\leq 0 \end{array}$$

*(ii) (Stability) The control objective of enclosing the TSat is guaranteed through* $u_{i}$ *in Equation (6), which is determined by* $u_{L}$ *in Equation (17) and* $u_{ci}^{*}$ *in Equation (21), namely the norm of the TT error* $\left\|x_{\mathrm{TL}}\right\|$ *and the CT errors* $\left\|{\tilde{x}}_{Li}\right\|$ *are both uniformly ultimately bound.*

## 4. Stability Analysis

The proof of Theorem 1 includes two parts.

### 4.1. Proof of the Optimality Results

Substituting Equations (20) and (21) into Equation (22), and Equation (22) can be rewritten as the following quadratic-like form:

(23) $$I_{i}^{s}=-\left[{\tilde{x}}^{T}\;{\left(\tilde{x}-\xi \right)}^{T}\right]\left(Z_{i}+\eta T_{i}\right)\left[\begin{array}{c} \tilde{x}\\ \tilde{x}-\xi \end{array}\right]$$

where $Z_{i}:R^{6N}\times R^{6N}\to R^{2\times (6N)\times 2\times (6N)}$ are written as

(24) $$Z_{i}=\frac{1}{2}\left[\begin{array}{cc} \Sigma_{i} & G_{i12}\\ G_{i12}^{T} & G_{i22} \end{array}\right]$$

with $\Sigma_{i}\left(\cdot \right)$ being defined in Definition 1. Meanwhile, we have

(25) $$\begin{array}{l} G_{i12}=\left(\sum_{j=1,j\neq i}^{N}\left(P_{i}+P_{j}\right)B_{j}{\hat{B}}_{j}^{T}\right)\left(R_{j}-\Phi_{j}\right)+\\ \;\left(\sum_{j=1,j\neq i}^{N}P_{j}\Delta B_{j}{\hat{B}}_{j}^{T}\right)\left(R_{j}-\Phi_{j}\right)-\\ \;{\left(A_{\mathrm{cl}}+{\hat{B}}_{i}\Delta B_{i}^{T}P_{i}\right)}^{T}\left(R_{i}-\Phi_{i}\right) \end{array}$$

(26) $$\begin{array}{l} G_{i22}=\sum_{l=1}^{N}{\left(R_{l}-\Phi_{l}\right)}^{T}{\hat{B}}_{l}{\hat{B}}_{l}^{T}\left(R_{l}-\Phi_{l}\right)+\\ \;2\sum_{j=1,j\neq i}^{N}{\left(R_{i}-\Phi_{i}\right)}^{T}\Delta B_{j}{\hat{B}}_{j}^{T}\left(R_{j}-\Phi_{j}\right)-\\ \;2\sum_{j=1,j\neq i}^{N}{\left(R_{i}-\Phi_{i}\right)}^{T}\Delta B_{i}{\hat{B}}_{i}^{T}\left(R_{i}-\Phi_{i}\right) \end{array}$$

where $A_{\mathrm{cl}}\left(\tilde{x}\right)≜A-\sum_{i=1}^{N}{\hat{B}}_{i}{\hat{B}}_{i}^{T}P_{i}\left(\tilde{x}\right)$.

As another part, $T_{i}:R^{6N}\times R^{6N}\to R^{2\times \left(6N\right)\times 2\times \left(6N\right)}$ is written as

(27) $$T_{i}=\frac{1}{2}\left[\begin{array}{cc} C_{i11} & C_{i12}\\ C_{i12}^{T} & C_{i22} \end{array}\right]$$

where

(28) $$\left\{\begin{array}{l} C_{i11}=\Psi_{i}\sum_{l=1}^{N}\Psi_{l}^{T}+\sum_{l=1}^{N}\Psi_{l}^{T}\Psi_{i}\\ C_{i12}=-\Psi_{i}\sum_{l=1}^{N}R_{l}^{T}-\sum_{l=1}^{N}\Psi_{l}R_{i}^{T}\\ C_{i22}=R_{i}\sum_{l=1}^{N}R_{l}^{T}+\sum_{l=1}^{N}R_{l}R_{i}^{T} \end{array}\right.$$

Since $\Psi_{i}\left(\tilde{x},\xi \right)$ defined in Equation (20) satisfies $\Psi_{i}\left(0,\xi \right)=0$, there exists neighborhoods around the origin $W_{i}$ which ensure $T_{i}$ is both semi-positive and definite. Noting $I_{i}^{s}={\left(I_{i}^{s}\right)}^{T}$ as an $I_{i}^{s}$ scalar function, consider the following quadratic-form polynomial.

(29) $$\begin{array}{l} Q_{i}^{s}=I_{i}^{s}+{\left(I_{i}^{s}\right)}^{T}\\ \;=-\left[{\tilde{x}}^{T}\;{\left(\tilde{x}-\xi \right)}^{T}\right]\cdot \\ \;\left(\left(Z_{i}+Z_{i}^{T}\right)+k\left(T_{i}+T_{i}^{T}\right)\right)\cdot \\ \;\left[\begin{array}{c} \tilde{x}\\ \tilde{x}-\xi \end{array}\right]\\ \;=2I_{i}^{s} \end{array}$$

Noting that each column vector of matrix $K={\left[I,0\right]}^{T}\in R^{2\times \left(6N\right)\times 6N}$ spans the kernel space of all $\left(T_{i}+T_{i}^{T}\right)$ at $\left(\tilde{x},\xi \right)=\left(0,0\right)$ and $K^{T}\left(Z_{i}\left(0,0\right)+Z_{i}^{T}\left(0,0\right)\right)K=\Sigma_{i}\left(0\right)$$+\Sigma_{i}^{T}\left(0\right)\geq 0$, there thus exists a constant $\bar{\eta }>0$ and a compact set $\Omega_{i}$ containing the origin, ensuring that $Q_{i}^{s}\leq 0$ for all $\eta \geq \bar{\eta }$ and $\left(\tilde{x},\xi \right)\in \Omega_{i}$, which implies that Equation (22) holds.

**Remark 7.** 
Equation (22) implies that there exists a nonnegative scalar function ${\overset{⌢}{e}}_{i}\left(\tilde{x},\xi \right)$ satisfying ${\overset{⌢}{e}}_{i}\left(\tilde{x},\xi \right)+I_{i}^{s}=0$, which further illustrates the group of CT controllers $\text{\{}u_{c1}^{*},\ldots ,u_{ci}^{*},\ldots ,u_{\mathrm{cN}}^{*}\text{\}}$ are approximate Nash equilibrium strategies for the differential game determined by Equations (9) and (11) according to Definition 3. Furthermore, noting that the auxiliary variable is tuned along the negative gradient of $V_{i}\left(\tilde{x},\xi \right)$, all CT controllers $u_{ci}^{*}$ drive the CT error $\tilde{x}$ to approach ξ, and $\left(\tilde{x},\xi \right)$ will finally converge to the manifold $M≜\left\{\left.\left(\tilde{x},\xi \right)\right|\tilde{x}-\xi =0\right\}$. As a result, $V_{i}\left(\tilde{x},\xi \right)\to V_{i}^{*}\left(\tilde{x}\right)$, which is the exact solution of the HJI PDE in Equation (13).

### 4.2. Proof of the Stability Results

Noting that $|e_{1l}(0)|<\pi /2$ and $|e_{2l}(0)|<\pi /2$ holds, there exists constants $B_{1l}<\pi /2$ and $B_{2l}<\pi /2$, ensuring $0\leq |e_{1l}(0)|<B_{1l}<\pi /2$ and $0\leq |e_{2l}(0)|<B_{2l}<\pi /2$. Considering an error vector $e={\left[e_{1}^{T},e_{2}^{T}\right]}^{T}\in R^{6}$, define compact set $\Omega_{\mathrm{TL}0}$ as follows:

(30) $$\Omega_{\mathrm{TL}0}=\underset{6-times}{\underset{︸}{\left[-B_{e},B_{e}]\times \ldots \times [-B_{e},B_{e}\right]}}$$

where constant $B_{e}≜\underset{r=1,2}{\max}\{B_{0},\arctan C_{r}\}$ with $B_{0}≜\underset{l=1,2,3}{\max}\{B_{1l},B_{2l}\}$ and positive constant $C_{r}$ being given later. Thus, $0<B_{e}<\pi /2$ always holds. According to the proposed transformation in Equation (15), the proof of $\left|q_{\mathrm{TL}l}-q_{\mathrm{TL}l,d}|<\tan(k_{1}(t)\pi /2\right)$ and $\left|{\dot{q}}_{\mathrm{TL}l}-{\dot{q}}_{\mathrm{TL}l}^{v}|<\tan(k_{2}(t)\pi /2\right)$ are equivalent to the proof of $e(t)\in \Omega_{\mathrm{TL}0}$, which implies the UUB of the TT error norm $\left\|{\tilde{q}}_{\mathrm{TL}}\right\|$.

Define an open set $\Omega_{e}$ as

(31) $$\Omega_{e}=\underset{6-times}{\underset{︸}{\left(-\frac{\pi }{2},\frac{\pi }{2})\times \ldots \times (-\frac{\pi }{2},\frac{\pi }{2}\right)}}$$

Thus, we have $\Omega_{\mathrm{TL}0}\subset \Omega_{e}$, and $e(0)\in \Omega_{e}$ holds. Due to the monotonicity of performance function $k_{r}(t)$ used in Equation (15), $k_{r}(t)<1$ for ∀t>0. Meanwhile, according to Equation (16), Equation (15) can be rewritten as follows:

(32) $$q_{\mathrm{TL}}=\tan(k_{1}(t)e_{1})+q_{\mathrm{TL},d}$$

(33) $$\begin{array}{l} {\dot{q}}_{\mathrm{TL}}=\tan(k_{2}(t)e_{2})+{\dot{q}}_{\mathrm{TL}}^{v}\\ \;=\tan(k_{2}(t)e_{2})+(-\lambda_{1}\tan e_{1}) \end{array}$$

In what follows, the boundedness of $e_{r}$ is given.

Step 1. Consider the following Lyapunov function:

(34) $$L_{1}=\frac{1}{2}\tan^{2}e_{1}$$

According to (15), the time derivative of Equation (34) is obtained.

(35) $${\dot{L}}_{1}=\frac{\tan^{T}e_{1}}{k_{1}\cos^{2}e_{1}}\left(-{\dot{k}}_{1}e_{1}+\frac{{\dot{q}}_{\mathrm{TL}}-{\dot{q}}_{\mathrm{TL},d}}{1+\tan^{2}\left(k_{1}e_{1}\right)}\right)$$

Substituting Equation (33) into Equation (35), we have

(36) $$\begin{array}{l} {\dot{L}}_{1}=\frac{\tan^{T}e_{1}}{k_{1}\cos^{2}e_{1}}\left(-{\dot{k}}_{1}e_{1}+\frac{{\dot{q}}_{\mathrm{TL}}-{\dot{q}}_{\mathrm{TL},d}}{1+\tan^{2}\left(k_{1}e_{1}\right)}\right)\\ \;\leq \frac{\left|\tan^{T}e_{1}\right|}{k_{1}\cos^{2}e_{1}}\left(-{\dot{k}}_{1}e_{1}-\frac{{\dot{q}}_{\mathrm{TL},d}}{1+\tan^{2}\left(k_{1}e_{1}\right)}+\frac{{\dot{q}}_{\mathrm{TL}}^{v}+\tan(k_{2}e_{2})}{1+\tan^{2}\left(k_{1}e_{1}\right)}\right)\\ \;\leq \frac{\left|\tan^{T}e_{1}\right|}{k_{1}\cos^{2}e_{1}}E_{1}(t){\dot{q}}_{\mathrm{TL}}^{v}+\frac{\left|\tan^{T}e_{1}\right|}{k_{1}\cos^{2}e_{1}}F_{1}(t) \end{array}$$

with

(37) $$\begin{array}{l} E_{1}(t)≜\frac{1}{1+\tan^{2}\left(k_{1}e_{1}\right)}\\ F_{1}(t)≜-\left|{\dot{k}}_{1}e_{1}\right|\frac{\left|\tan(k_{2}e_{2})\right|}{1+\tan^{2}\left(k_{1}e_{1}\right)}-\frac{{\dot{q}}_{\mathrm{TL},d}}{1+\tan^{2}\left(k_{1}e_{1}\right)} \end{array}$$

Note that $e_{1}$ and $e_{2}$ are bound on open set $\Omega_{e}$ for ∀t≥0. Since ${\dot{k}}_{1}$, $k_{2}$, and ${\dot{q}}_{\mathrm{TL},d}$ are all bound, the boundness of $\tan(k_{1}e_{1})$ and $\tan(k_{2}e_{2})$ can be obtained by Equation (31), which illustrates that $E_{1}(t)$ and $F_{1}(t)$ are all bound. There exist positive constants $c_{1,1}$, $c_{1,2}$, and $c_{1,3}$ that are as follows:

(38) $$0<c_{1,1}\leq \lambda_{\min}\left(E_{1}\right)\leq c_{1,2}$$

(39) $$0\leq \left\|F_{1}\right\|\leq c_{1,3}$$

where $\lambda_{\min}\left(E_{1}\right)$ is the minimum eigenvalue of matrix $E_{1}$. $\max\{F_{1l}\}$ is the maximum element of the vector $F_{1}$. Substituting Equation (16) into Equation (36) we have

(40) $$\begin{array}{l} {\dot{L}}_{1}\leq \frac{\tan^{T}\left|e_{1}\right|}{k_{1}\cos^{2}e_{1}}E_{1}(t){\dot{q}}_{\mathrm{TL}}^{v}+\frac{\tan^{T}\left|e_{1}\right|}{k_{1}\cos^{2}e_{1}}F_{1}(t)\\ \;\leq -\lambda_{1}c_{1,1}\frac{\tan^{2}\left|e_{1}\right|}{k_{1}\cos^{2}e_{1}}+c_{1,3}\frac{\tan^{T}\left|e_{1}\right|}{k_{1}\cos^{2}e_{1}}\left[\begin{array}{c} 1\\ 1\\ 1 \end{array}\right] \end{array}$$

Equation (40) illustrates that ${\dot{L}}_{1}<0$ if and only if $\tan\left|e_{1l}\right|>c_{1,3}/\lambda_{1}c_{1,1}$ for all l=1,2,3. Thus, we obtain the following:

(41) $$\max\left\{\left|e_{1l}\right|\right\}\leq C_{1}≜\max\left\{B_{0},\arctan\left(\frac{c_{1,3}}{\lambda_{1}c_{1,1}}\right)\right\}$$

Equation (41) illustrates that $\forall e_{1l}\in [-B_{e},B_{e}]\subset (-\pi /2,\pi /2)$ holds for ∀t≥0, namely $e_{1}$ can be always bound. Thus, the boundness of $q_{\mathrm{TL}}$ can be obtained by Equation (32). Moreover, ${\dot{q}}_{\mathrm{TL}}^{v}$ and ${\dot{q}}_{\mathrm{TL}}$ are bound by Equations (16) and (33).

Step 2. The following Lyapunov function is further considered.

(42) $$L_{2}=\frac{1}{2}\tan^{2}e_{2}$$

Similarly, by Equation (15), the time derivative of Equation (42) is

(43) $${\dot{L}}_{2}=\frac{\tan^{T}e_{2}}{k_{2}\cos^{2}e_{2}}\left(-{\dot{k}}_{2}e_{2}+\frac{{\ddot{q}}_{\mathrm{TL}}-{\ddot{q}}_{\mathrm{TL}}^{v}}{1+\tan^{2}\left(k_{2}e_{2}\right)}\right)$$

Note we have ${\ddot{q}}_{\mathrm{TL}}=H_{L}^{-1}u_{L}+h\left(q_{\mathrm{TL}},{\dot{q}}_{\mathrm{TL}}\right)$, where $h\left(q_{\mathrm{TL}},{\dot{q}}_{\mathrm{TL}}\right)≜-N_{\mathrm{TL}}\left(q_{\mathrm{TL}}\right)-\hat{C}\left(\omega \right){\dot{q}}_{\mathrm{TL}}$$-H_{T}^{-1}u_{T}$. $H_{L}≜m_{L}I_{3}$, $H_{T}≜m_{T}I_{3}$. Then Equation (43) can be rewritten as follows:

(44) $$\begin{array}{l} {\dot{L}}_{2}=\frac{\tan^{T}e_{2}}{k_{2}\cos^{2}e_{2}}\left(-{\dot{k}}_{2}e_{2}+\frac{{\ddot{q}}_{\mathrm{TL}}-{\ddot{q}}_{\mathrm{TL}}^{v}}{1+\tan^{2}\left(k_{2}e_{2}\right)}\right)\\ \;\leq \frac{\left|\tan^{T}e_{2}\right|}{k_{2}\cos^{2}e_{2}}\left(-{\dot{k}}_{2}e_{2}+\frac{H_{L}^{-1}u_{L}+h}{1+\tan^{2}\left(k_{2}e_{2}\right)}-\frac{{\ddot{q}}_{\mathrm{TL}}^{v}}{1+\tan^{2}\left(k_{2}e_{2}\right)}\right)\\ \;\leq \frac{\left|\tan^{T}e_{2}\right|}{k_{2}\cos^{2}e_{2}}E_{2}(t)u_{L}+\frac{\left|\tan^{T}e_{2}\right|}{k_{2}\cos^{2}e_{2}}F_{2}(t) \end{array}$$

where

(45) $$\begin{array}{l} E_{2}(t)≜\frac{H_{L}^{-1}}{1+\tan^{2}\left(k_{2}e_{2}\right)}\\ F_{2}(t)≜\frac{h+{\dot{q}}_{\mathrm{TL}}-{\ddot{q}}_{\mathrm{TL}}^{v}}{1+\tan^{2}\left(k_{2}e_{2}\right)}-\left|{\dot{k}}_{2}e_{2}\right| \end{array}$$

Note that $e_{1}$ is bound on $\Omega_{e}$ for ∀t≥0 by Step 1, and $e_{2}$ is bound on $\Omega_{e}$ for t=0. Since ${\dot{k}}_{1}$, $k_{2}$, h, and ${\dot{q}}_{\mathrm{TL},d}$ are all bound, the boundness of $\tan(k_{2}e_{2})$ can be obtained by Equation (31), which illustrates that $E_{2}(t)$ and $F_{2}(t)$ are all bound. There exist positive constants $c_{2,1}$, $c_{2,2}$, and $c_{2,3}$ that are as follows:

(46) $$0<c_{2,1}\leq \lambda_{\min}\left(E_{2}\right)\leq c_{2,2}$$

(47) $$0\leq \max\{F_{2l}\}\leq c_{2,3}$$

Considering the same definition in Step 1, substitute the TT controller $u_{L}$ by Equation (17) into Equation (44) and we have

(48) $$\begin{array}{l} {\dot{L}}_{2}\leq \frac{\tan^{T}\left|e_{2}\right|}{k_{2}\cos^{2}e_{2}}E_{2}(t)u_{L}+\frac{\tan^{T}\left|e_{2}\right|}{k_{2}\cos^{2}e_{2}}F_{2}(t)\\ \;\leq -\lambda_{2}c_{2,1}\frac{\tan^{2}\left|e_{2}\right|}{k_{2}\cos^{2}e_{2}}+c_{2,3}\frac{\tan^{T}\left|e_{2}\right|}{k_{2}\cos^{2}e_{2}}\left[\begin{array}{c} 1\\ 1\\ 1 \end{array}\right] \end{array}$$

Equation (48) illustrates that ${\dot{L}}_{2}<0$ if and only if $\tan\left|e_{2l}\right|>c_{2,3}/\lambda_{2}c_{2,1}$ for all l=1,2,3. Thus, we obtain the following:

(49) $$\max\left\{\left|e_{2l}\right|\right\}\leq C_{2}≜\max\left\{B_{0},\arctan\left(\frac{c_{2,3}}{\lambda_{2}c_{2,1}}\right)\right\}$$

Equation (49) illustrates that $\forall e_{2l}\in [-B_{e},B_{e}]\subset (-\pi /2,\pi /2)$ holds for ∀t≥0, namely $e_{2}$ can be always bound, and the boundness of the TT controller $u_{L}$ can be further obtained.

Based on all analyses above, Equations (41) and (49) illustrate that $e\in \Omega_{\mathrm{TL}0}\subset \Omega_{e}$ holds for ∀t≥0, which implies that $\Omega_{\mathrm{TL}0}$ is an invariant set of e. As a result, there exists an upper bound of the TT error norm $\varepsilon_{\mathrm{TL}}$, which can be user-defined by selecting proper parameters in $k_{1}(t)$, and it satisfies that $\left\|{\tilde{q}}_{\mathrm{TL}}\right\|<\left\|\tan\left(k_{1}(t)\pi /2\right){\left[\begin{array}{ccc} 1 & 1 & 1 \end{array}\right]}^{T}\right\|<\varepsilon_{\mathrm{TL}}$.

Step 3. According to Equation (6), all the CT error ${\tilde{x}}_{Li}$ satisfy Equation (9). Consider the following Lyapunov function.

(50) $$L_{3}=\sum_{i=1}^{N}V_{i}\left(\tilde{x},\xi \right)$$

Since $-\sum_{i=1}^{N}N\left(\left(\partial V_{i}/\partial \tilde{x}\right)H^{-1}{\hat{B}}_{i}{\hat{B}}_{i}^{T}H^{-T}{\left(\partial V_{i}/\partial \tilde{x}\right)}^{T}/2\right)-e_{i}\left(\tilde{x},\xi \right)/2\leq 0$, according to Equation (13), for all $\left(\tilde{x},\xi \right)\in \Omega_{i}$, $\left(\tilde{x},\xi \right)\neq \left(0,0\right)$, the time derivative of Equation (50) satisfies

(51) $$\begin{array}{l} {\dot{L}}_{3}\left(\tilde{x},\xi \right)=\sum_{i=1}^{N}\left(\frac{\partial V_{i}}{\partial \tilde{x}}\dot{\tilde{x}}\left(t\right)+\frac{\partial V_{i}}{\partial \xi }\dot{\xi }\left(t\right)\right)\\ \;=\frac{1}{2}\sum_{i=1}^{N}\left(-q_{i}(\tilde{x})-e_{i}(\tilde{x},\xi )\right)-\\ \;\frac{1}{2}\sum_{i=1}^{N}N\left(\frac{\partial V_{i}}{\partial \tilde{x}}{\hat{B}}_{i}{\hat{B}}_{i}^{T}{\frac{\partial V_{i}}{\partial \tilde{x}}}^{T}\right)\\ \;\leq -\frac{1}{2}\sum_{i=1}^{N}q_{i}(\tilde{x})<0 \end{array}$$

Equation (51) implies that all the CT controllers $u_{ci}^{*}$ given by Equation (21) ensure an asymptotic stability for the corresponding CT error ${\tilde{x}}_{Li}$ at the origin [39], namely ${\left.{\tilde{x}}_{Li}(t)\right|}_{t\to \infty }\to 0$.

So far, Theorem 1 has been proved.

## 5. Simulation Results

The simulation results of an example in-orbit surrounding task by one LSat and N=6 SSats to surround an escaping unknown TSat are shown. The orbit of the LSat is the same as [27]. The desired pursuit state of the LSat $x_{\mathrm{TL},\;d}={\left[q_{\mathrm{TL},\;d}^{T},{\dot{q}}_{\mathrm{TL},\;d}^{T}\right]}^{T}$ shown in Figure 1 is set as $q_{\mathrm{TL},\;d}={\left[-400,0,0\right]}^{T}$ m and ${\dot{q}}_{\mathrm{TL},\;d}=0$ m/s. Set the LSat mass $m_{L}=60$ kg. Set the TSat mass $m_{T}=300$ kg. Set SSat i mass $m_{i}=50$ kg. The actuator capability of the LSat and SSat i are, respectively, considered as $u_{Lmax}=180$ N and $u_{imax}=20$ N. The total time is 300 s, and the simulation step is set as 0.1 s.

**Figure 5:**
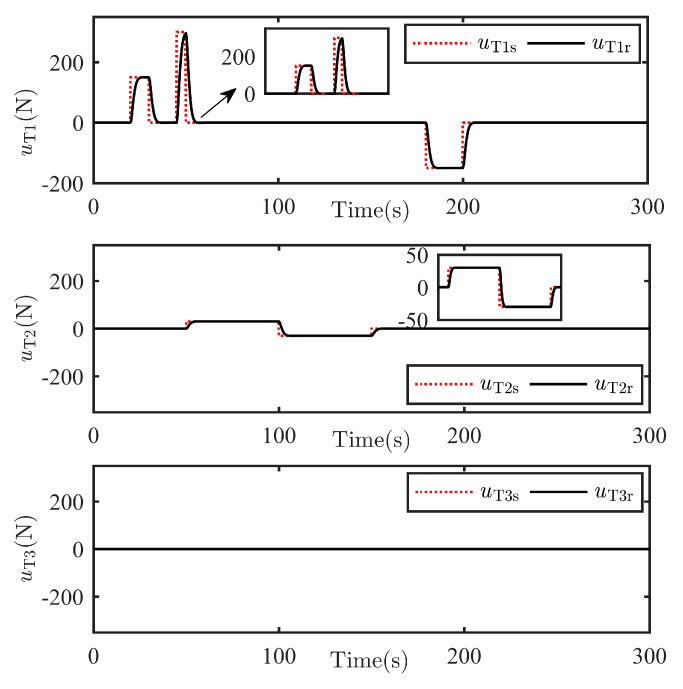
TSat escaping maneuver $u_{Tl}\left(t\right)$, l=1,2,3.

The TSat are considered as implementing the unknown escaping maneuver command $u_{T}\left(t\right)$, satisfying the following differential equation, and the maneuver command $u_{T}\left(t\right)$ is generated as shown in Figure 5.

(52) $$u_{Ts}\left(t\right)={\ddot{u}}_{T}\left(t\right)+1.8{\dot{u}}_{T}\left(t\right)+u_{T}\left(t\right)$$

The initialization of the TSat relative states are considered as $q_{\mathrm{TL}}(0)=[-900$$,-500,-500{]}^{T}$ m and ${\dot{q}}_{\mathrm{TL}}(0)={\left[-5,-5,-5\right]}^{T}$ m/s, and the desired TT vector is set as $q_{\mathrm{TL},d}={\left[-400,0,0\right]}^{T}$ m, ${\dot{q}}_{\mathrm{TL},d}={\dot{q}}_{\mathrm{TL}}^{v}$. The CT error ${\tilde{x}}_{Li}$ for all SSats are shown in Table 1. The performance functions $k_{1}(t)$ and $k_{2}(t)$ are set as $\mu_{1}=\mu_{2}=0.1$, $a_{1}=5\times 10^{-5}$ and $a_{2}=5\times 10^{-4}$. The parameters in Equations (16) and (17) are sat as $\lambda_{1}=0.02$ and $\lambda_{2}=4.5$. The parameters in Equations (7), (8) and (20) are $\kappa_{i}=0.08$, $h_{F}=1$, $h_{C_{g},i}=1$, $\alpha_{Fi}=1\times 10^{-3}$, $\gamma_{i}=8\times 10^{-4}$, and η=0.04. Set $R_{i}=diag\{0\ldots I_{\left(i\right)}\ldots 0\}$, and the initialization of ξ is set as follows:

(53) $$\xi =\left[\begin{array}{l} {\left[[5,0,0]m,[0,0,0]m/s\right]}^{T}\\ {\left[[-1,0,0]m,[0,0,0]m/s\right]}^{T}\\ {\left[[0,4,0]m,[0,0,0]m/s\right]}^{T}\\ {\left[[0,-1,0]m,[0,0,0]m/s\right]}^{T}\\ {\left[[0,0,5]m,[0,0,0]m/s\right]}^{T}\\ {\left[[0,0,-2]m,[0,0,0]m/s\right]}^{T} \end{array}\right]$$

Based on the proposed ACSB method for the TSat envelope modeling, the TSat specific structures are divided into several connected cube cells, and the parameters (including their positions $d_{c_{g}}$ and threat radius $r_{g}$ of their circumscribed spheres) are shown in Table 2. Noting that the position of each circumscribed sphere $q_{C_{g}}$ is a time-varying value that changes synchronously with the escaping target, the initial position of each circumscribed sphere can be obtained as $q_{C_{g}}(0)=d_{C_{g}}+q_{\mathrm{TL}}(0)$.

**Figure 6:**
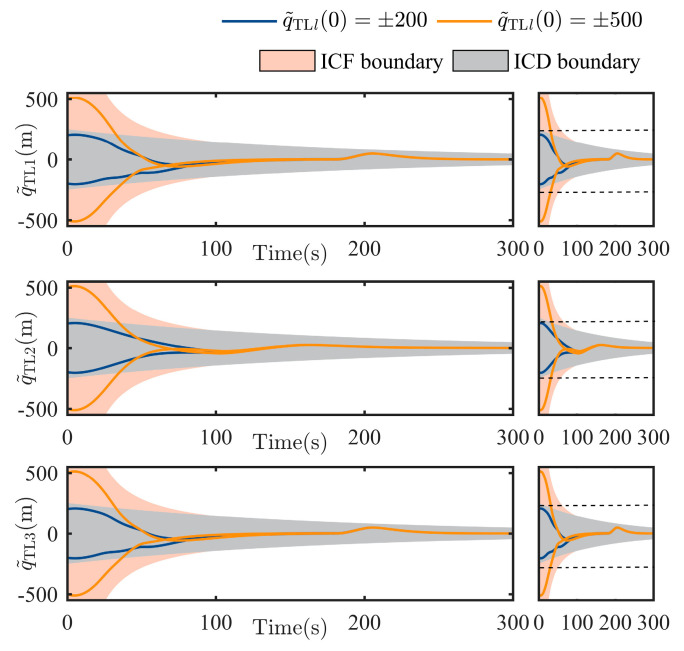
Transient behavior of the TT error ${\tilde{q}}_{TLl}\left(t\right)$, l=1,2,3.

**Figure 7:**
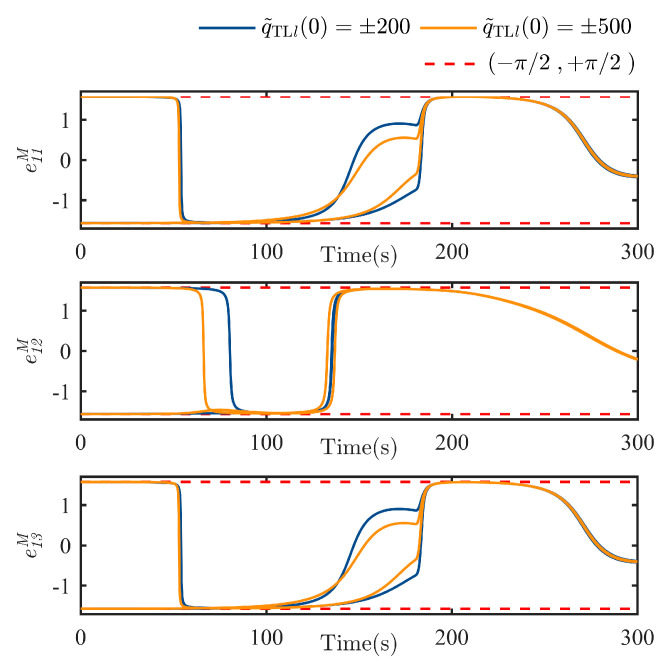
Transient behavior of the transformed TT error $e_{1l}\left(t\right)$, l=1,2,3.

**Figure 8:**
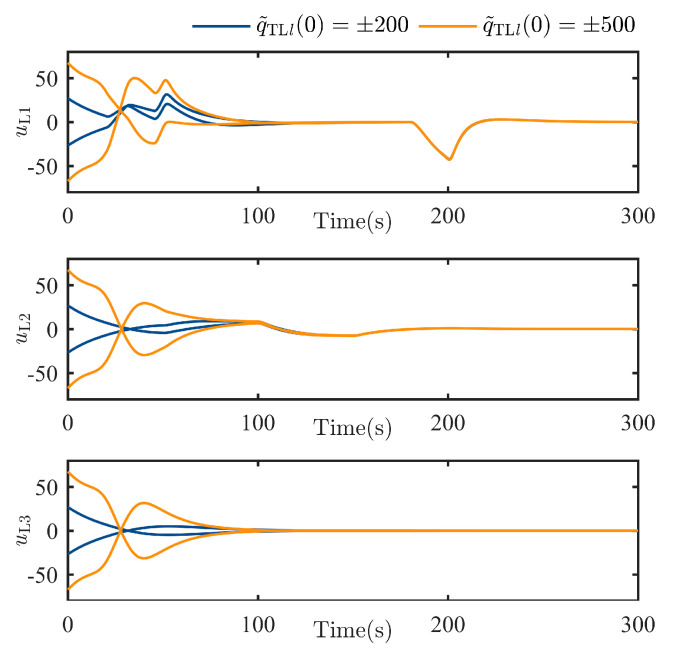
TT controller command $u_{Ll}\left(t\right)$, l=1,2,3.

For the TT part, by using the virtual controller and TT controller in Equations (16) and (17) respectively, the LSat tracking behavior against the TSat (including the dynamic response of the TT position error ${\tilde{q}}_{\mathrm{TL}}$, the transformed error $e_{1}$ and the target pursuit controller ($u_{L}$) are firstly shown. Note the initial conditions are commonly asked by the boundaries which can guarantee both user-defined transient and steady-state performance. To further illustrate the advantages of removing these conditions, two categories of boundaries, namely the initial-condition-free (ICF) boundaries and the initial-condition-dependent (ICD) boundaries, are compared under different initialization status. The initial TT states considered are shown in Table 3, and corresponding comparison results are shown in Figure 6, Figure 7 and Figure 8.

Thanks to the funnel-like boundaries constructed by the arctan-based transformation in Equation (15), as shown in Figure 6, the TT error in each channel under all initial status is successfully constrained by the funnel-like boundaries $\pm \tan(k_{1}(t)\pi /2)$ from t=0 to the end. Furthermore, noting that the boundaries have no intersections with y-axis at t=0 (see the gray aera in Figure 6), arbitrarily finite initial TT error will fall into the boundaries, which also verifies that the controller in Equations (16) and (17) work globally. A different situation happens to the ICD boundaries, namely only group 2 and 3 can be contained by the ICD boundaries. It illustrates that ICD boundaries will be failed for group 1 and 4, which implies the advantages of the proposed ICF boundaries. Meanwhile, by adjusting $a_{1}$ and $\mu_{1}$ in $k_{1}(t)$, the boundaries constructed by Equation (15) achieve a whole-process constraint with adjustable performance. As shown in Figure 6, considering the initialization and the parameters setting above, the tracking accuracy of the TT error can be shaped into [−1,1] m at 300 s as we prescribed.

Further in Figure 7, the responses in each transformed TT error channel $e_{1l}$ can be always kept in the open set (−π/2,π/2), which verifies the conclusion given in Equation (41), namely $\forall e_{1l}\in [-B_{e},B_{e}]\subset (-\pi /2,\pi /2)$ holds for ∀t≥0. According to the transformation given in Equation (15), the intersection between arbitrary $e_{1l}$ and the open set (−π/2,π/2) implies that the intersection between the corresponding TT error ${\tilde{q}}_{TLl}\left(t\right)$ and boundaries $\pm \tan(k_{1}(t)\pi /2)$.

The TT controller command in each channel $u_{Ll}\left(t\right)$ is shown in Figure 8, respectively. It illustrates that the controller designed in Equations (16) and (17) can firstly track the escaping behavior of the TSat successfully. On the other hand, the proposed controller will not ask high requirements for the actuators of the LSat (such as extremely high amplitude or sudden step), which also guarantees feasibilities for all SSats with a weaker maneuverability than the LSat.

**Figure 9:**
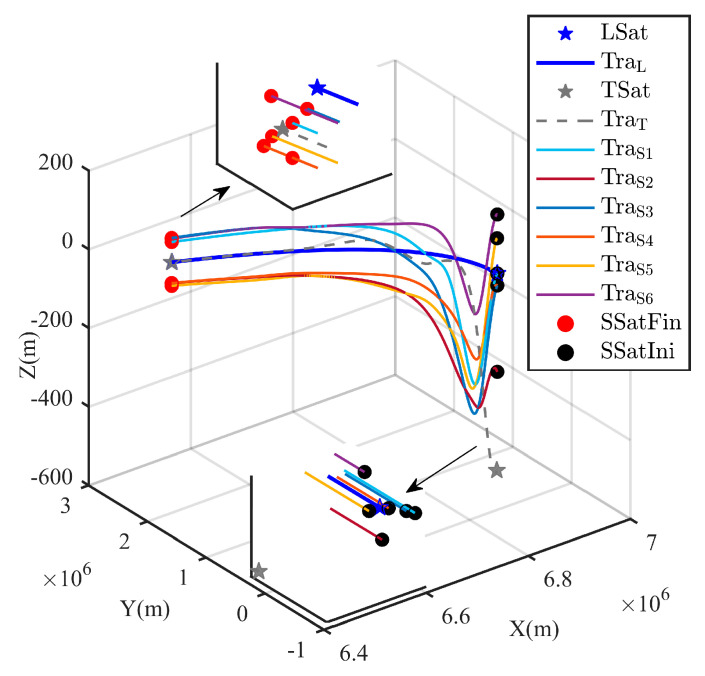
TSat surrounding trajectories in ECI frame.

**Figure 10:**
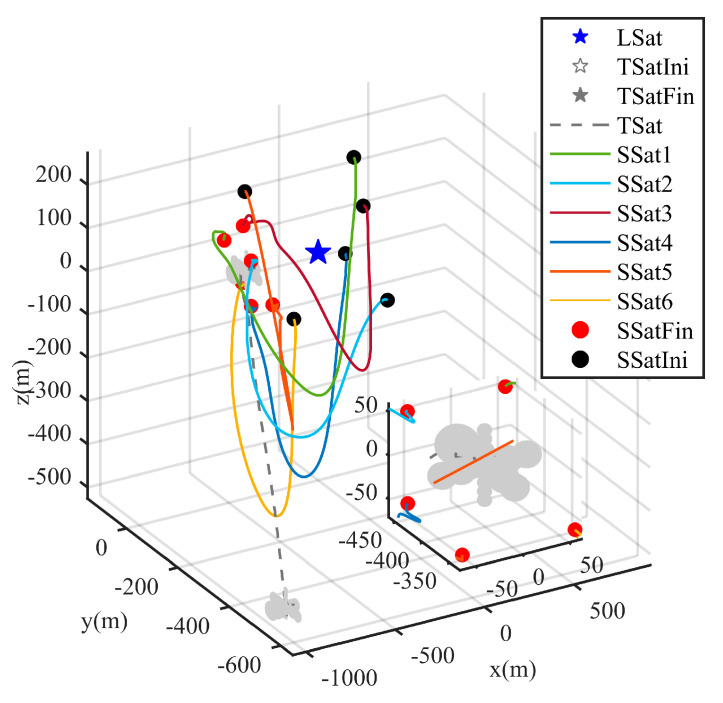
TSat surrounding trajectories in LVLH frame.

**Figure 11:**
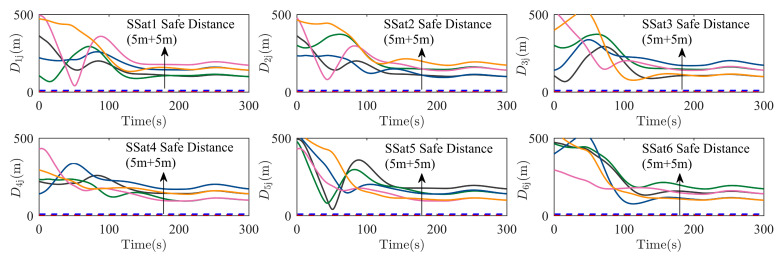
The distance between SSat i and each SSat j, j≠i, during the TSat surrounding process.

**Figure 12:**
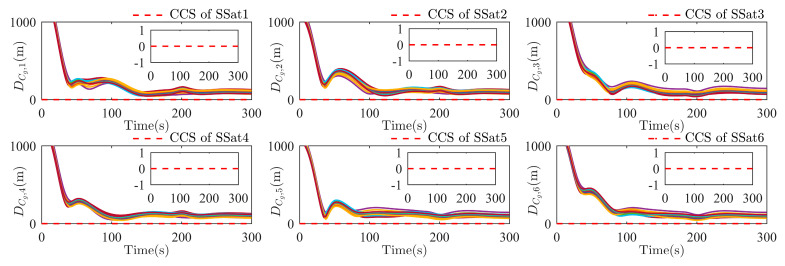
The distance between SSat i and all envelope sphere, g=1,…,q, during the TSat surrounding process, and the CCS for each SSat.

**Figure 13:**
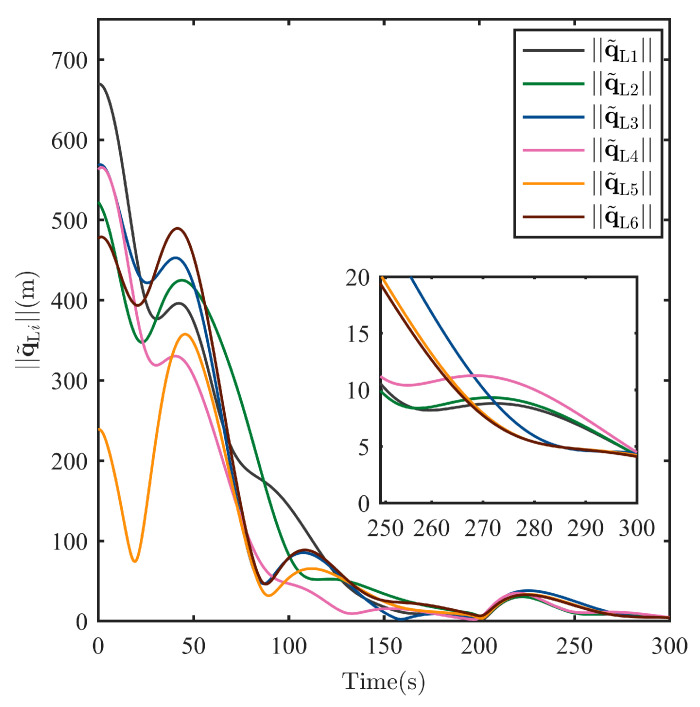
Norm of the surrounding CT error $\left\|{\tilde{q}}_{Li}\right\|$ of each SSat.

**Figure 14:**
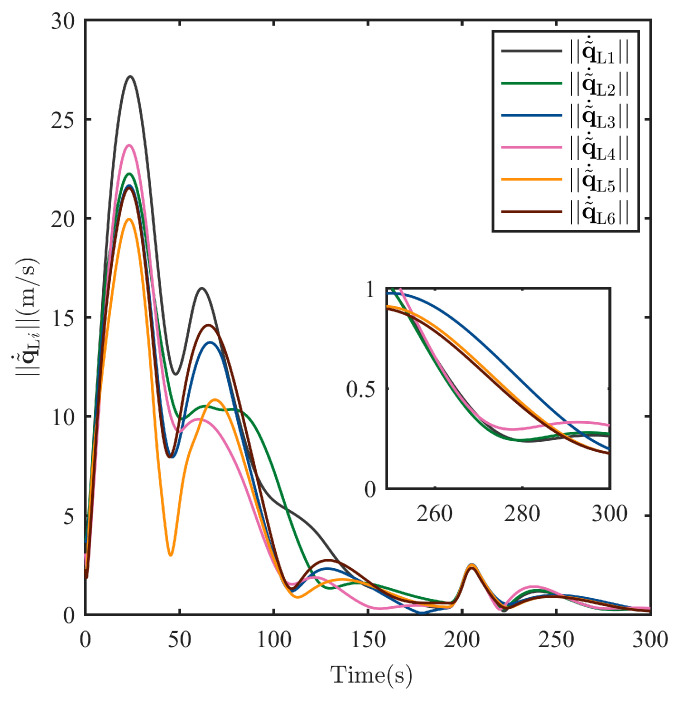
Norm of the surrounding CT error $\left\|{\dot{\tilde{q}}}_{Li}\right\|$ of each SSat.

**Figure 15:**
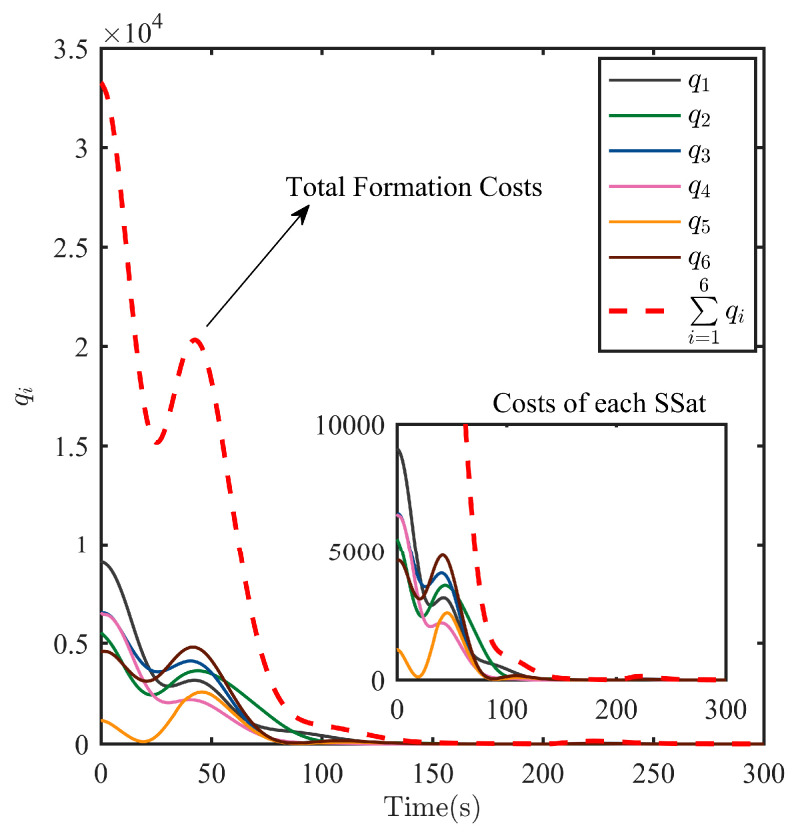
Behavior of each SSat running costs $q_{i}$ and their sum.

**Figure 16:**
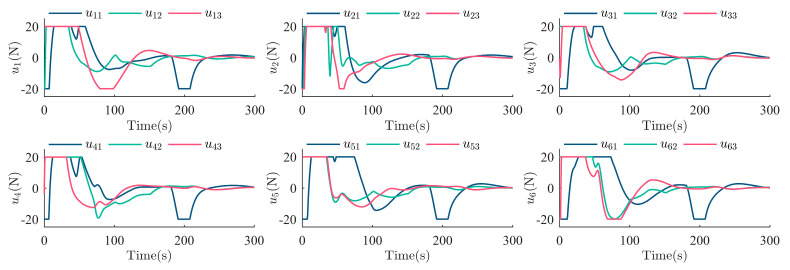
The control command $u_{i}={\left[u_{i1},u_{i2},u_{i3}\right]}^{T}$ of each SSat.

After the escaping unknown TSat tracking is guaranteed by the TT controller above, it is shared with all SSats as shown in Equation (6). Then, the Nash equilibrium strategies $u_{ci}^{*}$ for all SSats are given in Equation (21) with the immersed auxiliary variable ξ tuned by Equation (20) to ensure a safe surrounding of the escaping TSat with specific structures. Considering the initialization shown in Table 1 and the parameters set above. The process of the surrounding CT is shown in Figure 9, Figure 10, Figure 11, Figure 12, Figure 13, Figure 14, Figure 15 and Figure 16.

The whole process of the surrounding CT is shown in Figure 9 and Figure 10 with respect to ECI frame and the LSat LVLH frame respectively. Due to the orbit inclination of the LSat are set as 0 deg, clearer distance is shown on the z-axis in Figure 9. In particular, as shown in Figure 10, the control strategies $u_{i}$ in Equation (6) firstly guarantee the successful surrounding of the escaping TSat. Through the partial zoom in Figure 10, we can see that all the SSats get into the desired position around the TSat while any collision is avoided.

To further check the effectiveness of collision avoidance (including the collision between each SSat $D_{ij}$ with i,j=1,…,N, i≠j, and the collision between any SSat and the specific structures of the TSat $D_{C_{g},i}$ with g=1,…,q, i=1,…,N), the real-time distance between each SSat is shown in Figure 11, and the real-time distance from the ith SSat to all modeled structural envelope spheres is shown in Figure 12. As shown in Figure 11, the ith SSat can keep the safe distance during the whole surrounding process with the safe range of each SSat being 5 m, which implies that any collision between each SSat is avoided. Then, a collision checking signal (CCS) $SN_{CCS,i}$ ($SN_{CCS,i}≜\sum_{g=1}^{q}sn_{i,C_{g}}$ with $sn_{i,C_{g}}=1$ if and only if the ith SSat has a collision with the gth envelope sphere) is used in Figure 12 to show the effectiveness of the collision avoidance between all SSats and all envelope spheres. As the distance is reduced along the surrounding process, the CCSs of all SSats are always kept as 0, which illustrates the collision avoidance of the formation with respect to the specific structures of the TSat.

To show the surrounding CT accuracy, the real-time norm of the CT error (including the norm of the position error $\left\|{\tilde{q}}_{Li}\right\|$ and the velocity error $\left\|{\dot{\tilde{q}}}_{Li}\right\|$ for all i=1,…,N) are, respectively, shown in Figure 13 and Figure 14. Both the position tracking error and the velocity tracking error shows an asymptotic stability, and the final tracking accuracy at 300 s for position tracking and velocity tracking has reached, respectively, into $\left\|{\tilde{q}}_{Li}\right\|<5$ m and $\left\|{\dot{\tilde{q}}}_{Li}\right\|<0.5$ m/s.

As the most intuitive explanation of all results shown in Figure 9, Figure 10, Figure 11, Figure 12, Figure 13 and Figure 14, responds of the running costs $q_{i}\left(\tilde{x}\right)$ in Equation (12) and responds of the total running cost of the whole formation $\sum_{i=1}^{N}q_{i}$ are shown in Figure 15. From Figure 15, it is shown that both the total running cost $\sum_{i=1}^{N}q_{i}$ and the running costs of each SSat $q_{i}$ are ensured to be minimized. Note the change at 50 s is caused by the process that x≠ξ. As the total running cost $\sum_{i=1}^{N}q_{i}$ and the running costs of each SSat $q_{i}$ are minimized to zero, according to their definition given in Equation (12), all the CT error ${\tilde{x}}_{Li}$ satisfy that ${\tilde{x}}_{Li}\to 0$, and the collision threat of all SSats are minimized.

Figure 16 shows the controller command actually applied to each SSat according to Equation (6), which is mainly determined by the TT controller $u_{L}$ and the CT controller $u_{ci}^{*}$. As it is shown, the actuators of each SSat face saturation status at around 0 s–80 s, which is caused by the huge initial TT error and the initial CT error. Meanwhile, to synchronize with the LSat when the TSat is escaping, all SSats also update their control commands at around 100 s and 200 s.

To further support the advantages on CT performance, another collision-free controller against dynamic obstacles called cognitive-conditioned-reflex-based (CCRB) methods, similar to [44], is tested as a comparison under the same situation, namely the TT control command shared by the LSat, initialization of the formation, desired surrounding configuration, and the cube-based target modeling results are all considered to be the same as the OCSS method proposed. The compared CT performance includes the average total fuel consumption (ATFC), average minimum distance to obstacles (AMD_obs), and average minimum distance to satellites (AMD_sat). ATFC is calculated by the average value of six SSats of their total fuel consumption during the whole simulation time, namely 300 s. AMD_obs and AMD_sat, respectively, record the average minimum distance from each SSats to all obstacles and the average minimum distance from each SSats to each other during the whole 300 s. Figure 16 shows the surrounding path found for each SSats in LVLH frame by using the CCRB technique, and the CT performances are compared based on all the indices above in Table 4.

**Figure 17:**
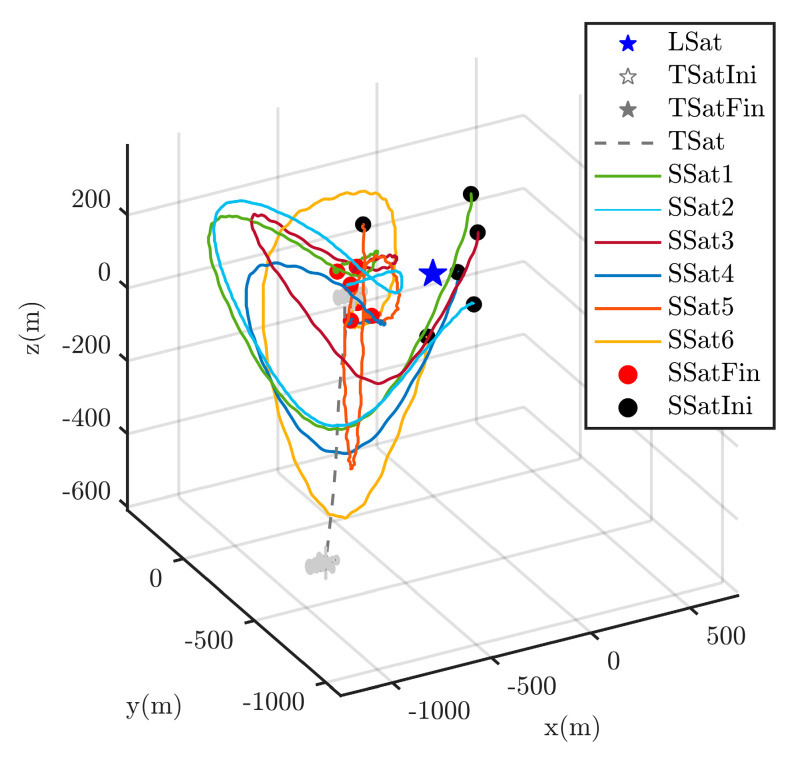
TSat surrounding trajectories in LVLH frame based on CCRB methods.

As shown in Figure 17 firstly, compared with the trajectories obtained by OCSS in Figure 10, the trajectories obtained by CCRB show chattering phenomenon due to its search & learning mechanism especially at the area closing to the target and its specific structures, which also result in a higher fuel consumption shown in Table 3. And as shown by the other indices, namely AMD_sat and AMD_obs, the results obtained by OCSS generally show a better performance than the one obtained by CCRB, which mainly thanks to the reference manifold ensuring the running costs descent along its gradient designed by Equation (20).

## 6. Conclusions

A safe huge-target surrounding control technique is proposed for SSat formation to achieve an optimal collision-avoidance FSC problem against a TSat with fully unknown dynamics and escape behaviors. Such a FSC problem is firstly divided into a TT sub-objective and a CT sub-objective. Funnel-like initial-condition-free boundaries with whole-process adjustable performances are proposed to directly constrain the TT error showing a prescribed response. Based on the ACSB modeling technique for collision threat description, the collision-avoidance CT problem is transformed into a nonzero-sum differential game and dynamic Nash equilibrium strategies for each SSat are constructed without iteration or learning calculations. In the future, we aim to solve the unknown disturbances and uncertainties that may occur in the FSC process.

## Figures and Tables

**Table 1:** Initial Relative State $x_{Li}$ and Desired Relative State $x_{Ti,d}$ of SSat i.

	$x_{Li}$	$x_{Ti,d}$
SSat 1	${\left[[200,0,200]m,[1,-2,-0.5]m/s\right]}^{T}$	${\left[[-50,50,50]m,[0,0,0]m/s\right]}^{T}$
SSat 2	${\left[[30,-250,0]m,[-2,3,1]m/s\right]}^{T}$	${\left[[-50,-50,50]m,[0,0,0]m/s\right]}^{T}$
SSat 3	${\left[[210,-30,100]m,[1,-3,2]m/s\right]}^{T}$	${\left[[50,50,50]m,[0,0,0]m/s\right]}^{T}$
SSat 4	${\left[[110,-30,0]m,[3,0.5,-0.5]m/s\right]}^{T}$	${\left[[-50,-50,-50]m,[0,0,0]m/s\right]}^{T}$
SSat 5	${\left[[-280,90,130]m,[0.5,-1,2]m/s\right]}^{T}$	${\left[[50,-50,-50]m,[0,0,0]m/s\right]}^{T}$
SSat 6	${\left[[80,150,-230]m,[2,0.5,-1]m/s\right]}^{T}$	${\left[[50,50,-50]m,[0,0,0]m/s\right]}^{T}$

**Table 2:** Initial position of the circumscribed spheres center $q_{C_{g}}$ and threat radius $r_{g}$.

Specific Structures	$d_{C_{g}}$	$r_{g}$
Core Cabin	${\left[0,0,0\right]}^{T}$m	$5\sqrt{2}$m
	${\left[-20,0,0\right]}^{T}$m	$15\sqrt{2}$m
	${\left[-50,0,0\right]}^{T}$m	$15\sqrt{2}$m
	${\left[-80,0,0\right]}^{T}$m	$15\sqrt{2}$m
Transporter	${\left[15,0,0\right]}^{T}$m	$10\sqrt{2}$m
	${\left[35,0,0\right]}^{T}$m	$10\sqrt{2}$m
	${\left[55,0,0\right]}^{T}$m	$10\sqrt{2}$m
Payload Cabin I	${\left[0,-10,0\right]}^{T}$m	$5\sqrt{2}$m
	${\left[0,-25,0\right]}^{T}$m	$10\sqrt{2}$m
	${\left[0,-45,0\right]}^{T}$m	$10\sqrt{2}$m
Payload Cabin II	${\left[0,10,0\right]}^{T}$m	$5\sqrt{2}$m
	${\left[0,25,0\right]}^{T}$m	$10\sqrt{2}$m
	${\left[0,45,0\right]}^{T}$m	$10\sqrt{2}$m
Solar Array I	${\left[0,0,-10\right]}^{T}$m	$5\sqrt{2}$m
	${\left[0,0,-20\right]}^{T}$m	$5\sqrt{2}$m
	${\left[0,0,-30\right]}^{T}$m	$5\sqrt{2}$m
	${\left[0,0,-40\right]}^{T}$m	$5\sqrt{2}$m
Solar Array II	${\left[0,0,10\right]}^{T}$m	$5\sqrt{2}$m
	${\left[0,0,20\right]}^{T}$m	$5\sqrt{2}$m
	${\left[0,0,30\right]}^{T}$m	$5\sqrt{2}$m
	${\left[0,0,40\right]}^{T}$m	$5\sqrt{2}$m

**Table 3:** Different TT error initialization status ($q_{\mathrm{TL}}(0)$, ${\dot{q}}_{\mathrm{TL}}(0)$, ${\tilde{q}}_{\mathrm{TL}}(0)$ and ${\dot{\tilde{q}}}_{\mathrm{TL}}(0)$) compared.

	$q_{\mathrm{TL}}\left(0\right)$	${\dot{q}}_{\mathrm{TL}}\left(0\right)$	${\tilde{q}}_{\mathrm{TL}}\left(0\right)$	${\dot{\tilde{q}}}_{\mathrm{TL}}\left(0\right)$
1	${\left[-900,-500,-500\right]}^{T}$	${\left[-5,-5,-5\right]}^{T}$	${\left[-500,-500,-500\right]}^{T}$	${\left[-5,-5,-5\right]}^{T}$
2	${\left[-600,-200,-200\right]}^{T}$	${\left[-2,-2,-2\right]}^{T}$	${\left[-200,-200,-200\right]}^{T}$	${\left[-2,-2,-2\right]}^{T}$
3	${\left[-200,200,200\right]}^{T}$	${\left[2,2,2\right]}^{T}$	${\left[200,200,200\right]}^{T}$	${\left[2,2,2\right]}^{T}$
4	${\left[100,500,500\right]}^{T}$	${\left[5,5,5\right]}^{T}$	${\left[500,500,500\right]}^{T}$	${\left[5,5,5\right]}^{T}$

**Table 4:** Surrounding CT performance between proposed OCSS and CCRB methods.

Methods	Indices	Value	
OCSS	ATFC	**75.12** (m/s)	
	AMD_sat	**83.3** (m)	
	AMD_obs	SSat 1	**88.21** (m)
		SSat 2	**88.49** (m)
		SSat 3	85.75 (m)
		SSat 4	**88.46** (m)
		SSat 5	**90. 81** (m)
		SSat 6	86.31 (m)
CCRB	ATFC	128.3 (m/s)	
	AMD_sat	82.41 (m)	
	AMD_obs	SSat 1	84.84 (m)
		SSat 2	87.09 (m)
		SSat 3	**88.27** (m)
		SSat 4	86.2 (m)
		SSat 5	86.15 (m)
		SSat 6	**90.29** (m)

## Data Availability

The original contributions presented in this study and all needed parameters are included in the article. Further inquiries can be directed at the corresponding author(s).
